# Balancing Prolificacy and Lamb Meat Production: Links Among Litter Size, Prenatal Development, Carcass Traits, and Meat Quality in Sheep

**DOI:** 10.3390/ani16193128

**Published:** 2026-10-06

**Authors:** Song Guan, Dexin Zhao, Haoliang Chai, Linfeng Zhou, Weiqi Peng, Hongzhi Wu

**Affiliations:** 1 Tropical Crops Genetic Resources Institute, Chinese Academy of Tropical Agricultural Sciences, Haikou 571101, China; 2 College of Animal Science and Technology, Northeast Agricultural University, Harbin 150030, China

**Keywords:** prolificacy, litter size, fetal programming, lamb survival, carcass traits, meat quality

## Abstract

Prolificacy can increase lamb output per ewe, but its productive value depends on the survival, growth, and carcass performance of each additional lamb. This review examines the pathway from genetic control of ovulation to realized litter size, prenatal development, neonatal viability, postnatal growth, carcass traits, and meat quality. Major fecundity genes, including *BMPR1B*, *BMP15*, *GDF9*, and *B4GALNT2*, alter ovulation rate, although evidence for direct effects of these loci on muscle development or meat quality remains scarce. Most phenotypic differences are more plausibly mediated through fetal number and the maternal environment. Compared with singletons, multiple-born lambs generally have less placental tissue per fetus, lower birth weight, reduced neonatal vigor, and slower preweaning growth. Prenatal restriction may alter myofiber formation, adipogenesis, and connective tissue development, but its later expression depends strongly on postnatal nutrition and slaughter endpoint. Across studies, litter size has a clearer and more consistent association with survival, growth trajectory, and carcass readiness than with ultimate pH, color, water-holding capacity, tenderness, intramuscular fat, fatty acid composition, or sensory quality. Reported contrasts vary with age, diet, carcass weight, and fatness; adjustment can attenuate some observed differences, but it may also remove effects mediated through growth. An effective production strategy therefore targets optimum rather than maximum prolificacy. Genetic selection should be integrated with pregnancy diagnosis, fetal-number-specific nutrition, colostrum management, rearing allocation, targeted finishing, and carcass feedback, with performance evaluated as saleable carcass output per ewe. The evidence is primarily observational for natural birth-type comparisons, while controlled nutritional models support selected prenatal mechanisms rather than equivalence to natural multiple pregnancy.

## Simple Summary

Increasing the number of lambs born per ewe can raise flock output, but a larger litter is useful only when the lambs survive and develop into marketable carcasses. Twins and triplets are usually lighter at birth because they share uterine space, placental supply, colostrum, and milk. The smallest lambs are therefore more vulnerable to cold, starvation, mismothering, and slow early growth. These disadvantages are most pronounced in triplet and larger litters, yet they do not mean that meat from multiple-born lambs is intrinsically poorer. When lambs are compared at a similar carcass weight and are managed under suitable feeding and slaughter conditions, differences in tenderness, color, water retention, fat content, and flavor are often limited or inconsistent. The main effect of litter size is usually on survival, growth rate, and the time needed to reach slaughter. Farmers can reduce these losses by scanning ewes for fetal number, feeding them according to pregnancy demand, safeguarding colostrum intake, fostering or artificially rearing surplus lambs when needed, and grouping lambs by weight after weaning. Breeding programs should likewise avoid selecting the litter size alone. The most useful goal is a manageable number of vigorous lambs that produces more saleable meat per ewe without unacceptable costs to animal welfare, labor, feed use, or product uniformity.

## 1. Introduction

### 1.1. Prolificacy and the Efficiency of Lamb Meat Production

Reproductive performance shapes both the biology and economics of sheep production. Lambs born and reared determine how much live weight and carcass output a ewe produces, but they also change feed demand, labor, replacement costs, and flock profit [1,2,3,4]. Maternal breeding programs have consequently used conventional genetic evaluation, prolific breeds, and alleles with large effects on ovulation rate and litter size [5,6,7]. An extra lamb, however, contributes little if it dies, grows inefficiently, or fails to meet the intended carcass specification. Litter size at birth is therefore only one point in the production chain, not a sufficient measure of reproductive success.

Conception, ovulation, embryo and fetal survival, neonatal survival, and growth to sale all contribute to flock output, although their effects are unequal. Shorten et al. found that embryo and lamb survival influenced total weight weaned per ewe exposed more strongly than a comparable change in ovulation rate [8]. Bioeconomic models similarly predict that larger litters may improve gross margin and reduce feed use per unit of carcass sold, provided that survival, growth, carcass value, and the costs of managing prolific ewes remain favorable [3,4]. Thus, breeding decisions should be directed toward sustainable output of healthy, marketable lambs rather than the highest attainable litter size.

### 1.2. Potential Tradeoffs and Synergies

Prolificacy creates biological pressure at the level of the individual lamb. With more fetuses, the resources available to each conceptus often decline, especially when maternal size, placental capacity, or nutrient supply is limiting. Multiple born lambs are usually lighter at birth than singletons, although breed, parity, ewe live weight, nutrition, and environment modify the difference [9,10,11,12]. Low birth weight compromises vigor, heat production, and access to colostrum and thereby raises early mortality risk [11,12,13,14]. Before weaning, milk shared among littermates can further restrict growth [10,15]. These individual penalties do not automatically translate into lower ewe productivity: two moderately lighter lambs may outweigh one heavy singleton at weaning, and some multiples compensate after weaning when feed supply is adequate. Population data show that early differences among birth types may narrow with age, but the extent of recovery depends on breed, management, and the basis of comparison [10,15].

Carcass and meat quality require the same distinction between individual and flock-level responses. Slow early growth may postpone target slaughter weight and alter the timing of muscle and fat deposition [15,16], but it does not consistently worsen ultimate pH, color, water-holding capacity, tenderness, intramuscular fat, or nutritional composition. Reported birth type effects vary because birth weight, rearing type, growth rate, slaughter age, and physiological maturity are interrelated [15,16]. These sources of variation must be separated before poorer meat quality can be attributed to prolificacy.

### 1.3. Conceptual Distinctions Among Prolificacy, Litter Size, and Birth Type

Clear terminology is essential when reproductive and meat production traits are considered together. Prolificacy is the reproductive characteristic or propensity of a ewe or population to produce multiple offspring. Its expression depends on ovulation rate, fertilization, embryo survival, fetal survival, and maternal genetic background [1,5,8]. Litter size describes the number of offspring present at a specified stage, most commonly at pregnancy scanning, birth, or weaning. Birth type classifies an individual lamb by the number of lambs delivered in its birth litter as a singleton, twin, triplet, or member of a larger litter. Rearing type describes its postnatal rearing group and may differ from birth type because of mortality, fostering, or artificial rearing [10]. Here, fetal number denotes the number of fetuses present at the time of assessment; litter size at birth counts lambs delivered, including those that do not survive, whereas number reared refers to lambs subsequently raised by a ewe or another rearing arrangement. These measures must be recorded at their respective time points rather than inferred from one another. Thus, prolificacy denotes reproductive propensity, whereas fetal number, litter size at birth, birth type, and rearing type describe counts or classifications at defined stages. Observed litter size is an indicator of realized prolificacy, not an interchangeable definition of the underlying characteristic.

These terms are related but are not interchangeable. A lamb born as a twin need not carry alleles associated with high prolificacy, and the performance of a prolific breed cannot be attributed solely to its reproductive genotype. Most reported associations between litter size and lamb growth reflect a combination of maternal capacity, the intrauterine environment, birth weight, milk availability, and management [9,10,11,12,15]. Direct effects of prolificacy-associated alleles on skeletal muscle development, carcass composition, or meat quality remain insufficiently established. Here, a direct effect refers specifically to a genotype-attributable effect on lamb growth, carcass composition, or meat quality that is not mediated through fetal number or realized litter size. It does not refer simply to an allele that directly increases ovulation rate. Separating these pathways is a central requirement of this review.

### 1.4. Scope and Objectives

This review follows the biological sequence by which genetic potential for prolificacy becomes a realized litter and may subsequently affect prenatal development, neonatal survival, growth, carcass traits, and lamb meat quality. Emphasis is placed on skeletal muscle, adipose tissue, and connective tissue development, together with the reasons that published estimates differ. Birth type, rearing type, maternal nutrition, breed, and slaughter endpoint are considered explicitly.

The central question is not whether a twin is lighter than a singleton at one age, but whether a reproductive strategy yields more viable, saleable lamb meat per ewe. The framework follows lambs from birth through survival and rearing to sale, while accounting for ewe health and longevity, intervention frequency, and labor requirements. These outcomes connect reproductive performance with sustainable carcass output, meat quality, welfare, and management feasibility.

## 2. Materials and Methods

This narrative review integrates evidence on the relationships among prolificacy, realized litter size, prenatal development, lamb survival, growth, carcass traits, and meat quality in sheep. Two authors conducted the literature searches through PubMed (https://pubmed.ncbi.nlm.nih.gov/) and examined the reference lists of relevant publications. PubMed was searched using combinations of terms related to prolificacy, litter size, birth type, rearing type, maternal nutrition, lamb survival, carcass traits, and meat quality. The search was last updated on 26 September 2026. Differences in judgments about the relevance of publications were resolved by reassessing their content against the scope and objectives of the review. Formal counts of records retrieved and screened were not recorded.

The literature selection was guided by relevance to the review’s scope, with priority given to peer-reviewed original studies and reviews. Ovine studies provided the principal evidence for species-specific conclusions. Relevant genetic studies, observational birth-type comparisons, controlled nutritional and developmental experiments, and production studies were considered alongside reviews and foundational publications. Studies reporting intermediate outcomes, such as ovulation rate, placental function, birth weight, or survival, were retained even when carcass or meat-quality measurements were unavailable. Broader reviews and the mechanistic literature covering other species informed general biological explanations but were not treated as direct evidence of litter-size effects in sheep. Publications unrelated to the processes or outcomes covered by the review were not included. Multiple reports from the same study were considered together, retaining complementary findings without treating them as independent evidence. The final bibliography comprises 134 references published between 1986 and 2026. This range reflects the publication years of the cited literature rather than a predefined search restriction, and the references do not represent 134 independent experimental studies.

Evidence was synthesized qualitatively along the biological sequence outlined above. Fetal number, litter size at birth, and rearing type were distinguished, as were individual lamb performance and productive output per ewe. Findings from natural multiple pregnancies were considered separately from those obtained in models of maternal nutrient restriction, placental insufficiency, or low birth weight. These models informed possible developmental mechanisms but were not assumed to reproduce the effects of naturally occurring twin or triplet pregnancies. Associations between birth type and production traits were also distinguished from potential genotype-attributable effects not mediated through litter size, including those involving alleles at *BMPR1B*, *BMP15*, *GDF9*, and *B4GALNT2*. Direct evidence concerning carcass and meat-quality outcomes was distinguished from mechanistic observations supporting biological plausibility. Conflicting findings were interpreted in relation to study design, breed, sex, maternal nutrition, rearing conditions, sample size, the handling of dependence among lambs from the same litter or ewe, slaughter endpoint, and postmortem methods. Comparisons at the same age, live weight, carcass weight, or degree of fatness were interpreted separately because they address different biological questions. Small or unbalanced groups and variation in outcome definitions were considered when assessing the consistency and applicability of findings. No formal systematic screening process, study-level risk-of-bias assessment, or meta-analysis was undertaken. The conclusions should therefore be interpreted in light of potential selection bias, incomplete literature coverage, and heterogeneity among the studies reviewed.

**Figure 1:**
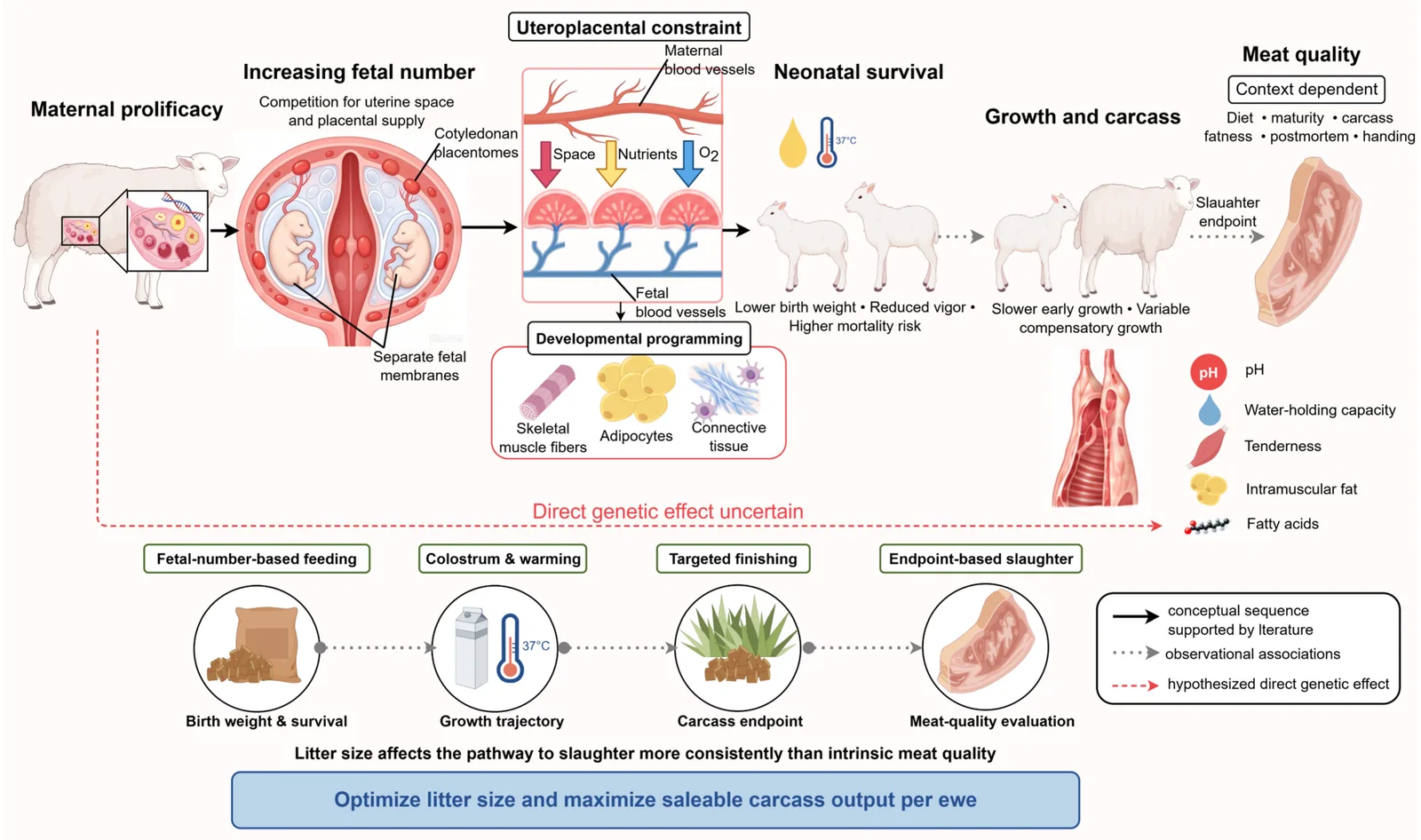
Integrated conceptual framework linking maternal prolificacy with prenatal development, lamb survival, growth, carcass performance, and meat quality. Maternal genetic potential, ovulation rate, embryo survival, nutrition, and environmental conditions jointly determine realized litter size. In multiple pregnancies, each fetus develops within a separate fetal membrane compartment and is connected through its umbilical and chorioallantoic vasculature to multiple cotyledonary placentomes distributed along the uterine wall. As fetal number increases, competition for uterine space and placental nutrient and oxygen supply may reduce birth weight and increase within-litter variation. These prenatal constraints can influence skeletal muscle, adipose tissue, and connective tissue development and subsequently affect neonatal vigor, thermoregulation, colostrum acquisition, survival, early growth, compensatory growth, slaughter readiness, and carcass performance. Effects on meat quality are less consistent and depend strongly on diet, physiological maturity, carcass fatness, slaughter endpoint, and postmortem handling. Direct pleiotropic effects of prolificacy-associated alleles on carcass and meat-quality traits remain insufficiently established. Fetal-number-based nutrition, colostrum and thermal support, targeted finishing, and endpoint-based slaughter can reduce biological risk and production variability. The overall objective is to optimize litter size and sustainable saleable carcass output per ewe, accounting for lamb survival and rearing, ewe health and longevity, intervention frequency, labor, and available farm resources. Black solid arrows depict the conceptual sequence supported by ovine evidence, gray dotted arrows depict associations that depend on growth and management, and the red dashed arrow denotes a hypothesized genotype-attributable effect not mediated through litter size, which has not been established for carcass or meat-quality traits. The arrows do not quantify effect size or prove that every intermediate link is causal. The terms fetal number, birth type, and rearing type are defined in Section 1.3. Created with Figdraw (https://www.figdraw.com, accessed on 26 September 2026).

Figure 1 was created using Figdraw (https://www.figdraw.com, accessed on 26 September 2026) to illustrate the conceptual relationships synthesized in this review. The figure is a schematic interpretation of published evidence. Its arrows do not quantify effect sizes or establish that every intermediate relationship is causal.

## 3. From Genetic Prolificacy to Realized Litter Size

### 3.1. Biological Components and Definitions of Prolificacy

Prolificacy is often represented by the number of lambs born per ewe lambing, but the observed litter size is the outcome of several successive biological processes. These include ovulation, fertilization, embryo survival, fetal survival, and successful parturition. Variation at any one of these stages can alter the number of lambs delivered, even when the underlying genetic potential for ovulation is similar [1,5,8]. The number of lambs weaned introduces further variation because it also reflects neonatal survival, maternal behavior, milk production, disease, and flock management. Litter size at birth and litter size at weaning should therefore be treated as related but distinct traits.

The distinction matters when prolificacy is linked with lamb growth or meat production. Selection for ovulation rate may increase the number of ova released without producing a corresponding increase in viable lambs. Likewise, a ewe that gives birth to three lambs may wean fewer kilograms of lamb than a ewe that rears two vigorous lambs. Prolificacy is consequently better viewed as a composite maternal trait rather than a simple count. Its productive value depends on how reproductive potential is converted into viable offspring and how efficiently those offspring develop to slaughter.

### 3.2. Major Genes and Polygenic Control

A small number of mutations with large effects have provided much of the current understanding of the genetic regulation of ovulation rate in sheep. The best characterized loci are *BMPR1B*, *BMP15*, *GDF9*, and *B4GALNT2*. The first three encode components of the transforming growth factor beta signaling system and regulate follicular recruitment, granulosa cell function, and oocyte development [5,6,7,17,18]. The *B4GALNT2* locus acts through a different mechanism involving ovarian expression of a glycosylation enzyme and altered glycosylation of proteins involved in follicular regulation [19]. The principal loci, representative alleles, and evidence concerning lamb production traits are summarized in Table 1. The FecB mutation in *BMPR1B* produces a largely additive increase in ovulation rate, and homozygous ewes generally remain fertile [7]. Mutations in the X-linked *BMP15* gene show more variable patterns. Several loss-of-function alleles increase ovulation rate in heterozygous ewes but cause ovarian failure and sterility in homozygous carriers [17,18]. Related mutations in *GDF9* can produce a similar dosage-dependent response [18]. This contrast has direct implications for breeding programs. Increasing the frequency of a favorable allele may improve litter size, but the outcome depends on the inheritance pattern, allele dosage, genetic background, and reproductive management of the flock. The consequences of a carrier genotype also depend on whether the increased ovulation rate yields more viable lambs or shifts the litter distribution toward births that exceed maternal or management capacity; for some *BMP15* and *GDF9* alleles, homozygous sterility creates an additional breeding risk [17,18]. At the flock level, genotype affects ovulation rate and the resulting litter-size distribution, which in turn influences birth weight and survival. The surviving lambs determine rearing and intervention requirements, while pregnancy and lactation demands affect ewe body condition and subsequent reproductive capacity. These linked responses determine the number of lambs reared and marketed per ewe. Higher ovulation therefore need not produce a proportional increase in marketable output, particularly when feed supply, maternal capacity, labor, or ewe longevity become limiting [3,4,8].

These major genes do not explain all variation in prolificacy. Most commercial populations show continuous variation in reproductive performance, and genome-wide association studies have identified numerous candidate regions related to follicular development, endocrine signaling, ovarian function, and embryo survival [20,21]. Many reported associations are specific to a breed or population and have not been independently validated. Litter size should therefore not be treated as a trait controlled by a small universal set of fecundity genes. In many populations, it remains a complex trait shaped by numerous variants with individually small effects, together with age, parity, body condition, season, nutrition, and management.

### 3.3. Direct, Maternal, and Permanent Environmental Effects

The phenotype of a lamb reflects more than its own genotype. Birth weight and early growth are influenced by direct genetic effects from the lamb, maternal genetic effects expressed through the ewe, permanent environmental effects associated with the dam, and temporary conditions during a particular pregnancy or lactation [2,10,22,23]. Common litter effects may also be substantial because lambs from the same pregnancy share the uterine environment, birth type, dam, and early management. These components are particularly important in prolific flocks. An allele carried by the ewe may increase ovulation rate and thereby alter the uterine environment experienced by her offspring. The same allele may or may not be inherited by an individual lamb. A difference observed between singleton and twin lambs can therefore arise without any direct effect of the lamb genotype. Conversely, an association between a lamb genotype and growth may be confounded if the genotype is also associated with the litter size of its dam. Genetic models that omit maternal or common-litter effects may assign environmental variation to the direct breeding value of the lamb and overestimate the opportunity for selection.

The relative importance of these effects changes with age. Maternal influences are usually the strongest for birth weight and early growth, when fetal nutrient supply and milk production dominate the lamb environment. Direct genetic effects tend to become more evident after weaning as the lamb becomes less dependent on the dam [23]. Accurate recording of dam identity, birth type, rearing type, parity, and fostering is therefore essential. Errors in these records can change estimated breeding values and reduce the accuracy of comparisons among sires or maternal lines [10].

### 3.4. Genetic Relationships with Lamb Survival, Growth, and Carcass Traits

Litter size generally has low heritability, although sufficient genetic variation exists to support gradual selection [22,23,24]. Low heritability does not imply that the trait lacks economic or biological importance. It indicates that a large proportion of observed variation arises from temporary environmental conditions and nonadditive biological processes. Repeated reproductive records from the same ewe can improve the accuracy of genetic evaluation. Published estimates of genetic relationships between litter size and lamb growth are not consistent across breeds. Some studies have reported favorable genetic correlations between ewe prolificacy and lamb weight, whereas others have found weak or unfavorable relationships [22,23,24]. This variation probably reflects differences in breed background, production environment, trait definition, and statistical model. It also reflects whether growth is expressed as individual lamb weight or total litter weight. Selection based only on individual weaning weight may favor heavier singleton lambs, whereas selection based on total litter weight may favor ewes that rear multiple lambs successfully.

Lamb survival has both direct and maternal genetic components, but their heritabilities are usually low [25]. The practical response to selection may therefore be slower than the response for growth or carcass traits. Nevertheless, even small improvements in survival can have substantial value in prolific flocks because each additional fetus creates both an opportunity for greater output and a risk of loss. Balanced breeding programs should consider litter size, survival, maternal ability, and lamb growth together rather than expecting improvement in one trait to produce automatic improvement in the others. Evidence concerning carcass traits is more limited. Birth type is commonly fitted as a systematic environmental effect in genetic evaluations of live weight, muscle depth, and fat depth, but this adjustment does not establish a genetic relationship between prolificacy and carcass composition [2,15]. Even less information is available for eating quality, intramuscular fat, fatty acid composition, or flavor. The absence of such evidence should not be interpreted as proof that no relationship exists. It shows that reproductive and meat quality traits have rarely been measured within the same pedigreed or genotyped populations.

### 3.5. Direct and Litter Size Mediated Effects on Lamb Phenotype

Two pathways should be distinguished when prolificacy is related to lamb production. The first is an indirect pathway in which the maternal genotype alters ovulation rate and realized litter size. The resulting fetal number affects placental development, nutrient supply, birth weight, milk allocation, and postnatal growth. This pathway is supported by substantial evidence and is likely to explain much of the observed difference between singleton and multiple-born lambs. The second is a potential genotype-attributable pathway in which an allele inherited by the lamb affects skeletal muscle, adipose tissue, growth, or metabolism without mediation through fetal number or realized litter size. This possibility is biologically plausible because members of the transforming growth factor beta superfamily participate in several developmental processes. Current evidence is nevertheless inadequate to establish such pleiotropic effects in sheep. Studies that compare prolific and nonprolific breeds without controlling for population structure, maternal genotype, litter size, and management cannot resolve the two pathways. Natural differences in litter size provide mainly observational evidence for the first pathway, whereas nutritional restriction and placental-insufficiency experiments test selected mechanisms under imposed conditions. Neither source of evidence, by itself, isolates a genotype-attributable effect on growth, carcass composition, or meat quality that is not mediated through litter size.

Future studies need to genotype both dams and offspring and include sufficient numbers of lambs from different birth types within each genotype. Embryo transfer, reciprocal mating, cross-fostering, and genomic maternal-effect models could further separate direct inheritance from the uterine and rearing environment. Until such evidence becomes available, differences in lamb growth, carcass value, or meat quality should be described primarily as consequences associated with realized litter size rather than as direct effects of prolificacy genes.

## 4. Prenatal Development of Multiple Born Lambs

### 4.1. Uterine Capacity, Placental Function, and Fetal Nutrient Supply

The relationship between litter size and lamb birth weight is not linear. Total litter weight generally increases as fetal number rises, but the gain becomes progressively smaller for each additional fetus. The average weight of each lamb therefore declines from singleton to twin and higher order births [9,26,27]. This response reflects the interaction between the intrinsic growth capacity of the fetus and the finite capacity of the maternal reproductive system. It should not be interpreted as evidence that every multiple pregnancy is pathologically growth restricted. Many twin pregnancies are supported successfully, particularly in mature ewes with adequate body reserves and placental development. Constraints become more apparent when fetal number exceeds the capacity of the ewe or when poor nutrition, young maternal age, disease, heat stress, or limited uterine space reduces the resources available to each fetus. The ovine placenta is cotyledonary, and maternal caruncles join fetal cotyledons to form placentomes. These structures provide the main sites for the exchange of oxygen, glucose, amino acids, fatty acids, and metabolic waste. Fetal growth depends on placental mass, placentome number and morphology, vascular development, blood flow, nutrient transporter activity, and the metabolic demands of the placenta itself [28,29]. Placental mass alone is therefore an incomplete measure of function. A relatively small placenta may support normal fetal growth when vascularity and nutrient transfer per unit of tissue are high, whereas a larger placenta may remain inadequate if blood flow or transporter activity is impaired.

Litter size alters both the amount and distribution of placental tissue. Multiple pregnancies can have greater total placental mass than singleton pregnancies, but the amount of placental tissue available to each fetus is usually lower. Dwyer and colleagues found that breed, litter size, and parity affected placental weight and placentome number, which were in turn related to birth weight and neonatal behavioral development [28]. These observations indicate that placental adaptation can partially accommodate additional fetuses but may not fully preserve nutrient supply to every member of a large litter. Placental competition is also uneven within a litter. Differences in implantation site, placentome development, vascular access, fetal sex, and umbilical blood flow may create substantial variation among littermates. Mean litter size consequently provides limited information about the nutritional history of an individual fetus. Litter weight, variation in birth weight within the litter, placental characteristics, and the position of each fetus should be recorded whenever possible.

### 4.2. Fetal Growth, Endocrine Adaptation, and Nutrient Sensing

When nutrient or oxygen delivery becomes limiting, fetal growth is adjusted through coordinated endocrine and metabolic responses. Circulating insulin and insulin-like growth factors support tissue growth and protein synthesis, whereas glucocorticoids and catecholamines help redistribute substrates during nutritional or hypoxic stress. Reduced insulin and insulin-like growth factor signaling can limit glucose utilization and anabolic activity in skeletal muscle. Increased catecholamine concentrations can preserve nutrients for organs with immediate survival priority but may further restrict peripheral tissue growth [30,31]. The timing of the constraint is important. Early gestation includes implantation, placental establishment, and the commitment of progenitor cells to major tissue lineages. Mid-gestation overlaps with rapid placental development and secondary myogenesis. Late gestation is characterized by substantial fetal weight gain and rapid hypertrophy of existing muscle fibers. Nutritional restriction during these periods may therefore produce different outcomes even when birth weight appears similar. Restriction during early or mid-gestation can alter placental development and the number of cells available for later tissue growth. Restriction during late gestation may have a more immediate effect on fetal weight and muscle fiber size [30,32,33].

Cellular nutrient sensing contributes to these responses. The mechanistic target of rapamycin pathway promotes protein translation, myoblast proliferation, and tissue growth when amino acids, energy, insulin, and growth factors are available. Maternal nutrient restriction in sheep has been associated with reduced phosphorylation of mechanistic target of rapamycin and ribosomal protein S6 in fetal muscle, together with fewer secondary muscle fibers [30]. Energy stress may also modify AMP-activated protein kinase activity, although responses differ with gestational stage, tissue, and severity of restriction. These pathways provide plausible links between limited placental supply and reduced muscle growth, but they should not be presented as mechanisms proven specifically in naturally conceived triplet or quadruplet pregnancies.

### 4.3. Programming of Skeletal Muscle Development

Skeletal muscle development has particular importance for meat production because the number of muscle fibers is largely established before birth. Postnatal muscle growth occurs mainly through enlargement of existing fibers. A persistent reduction in fiber number can therefore constrain later muscle mass even when the lamb receives adequate nutrition after birth [30,32,34]. Primary muscle fibers form early and provide a framework for the subsequent formation of secondary fibers. Secondary myogenesis accounts for most of the variation in final fiber number and occurs during a period when placental demand and fetal growth are increasing. Nutritional disturbance before or during secondary myogenesis can alter myoblast proliferation, differentiation, and fusion. In sheep, maternal restriction before muscle differentiation altered muscle fiber development in newborn lambs, while restriction during early to mid-gestation reduced secondary fiber formation and muscle fascicle area in fetuses [30,32]. More recent work has shown that poor maternal nutrition changes the abundance of myogenic progenitor cells and the expression of genes involved in cell proliferation, protein synthesis, and muscle function [33].

The response is not uniform across muscles. Muscles differ in developmental timing, contractile function, fiber type, and metabolic profile. A nutritional effect detected in the longissimus muscle may not occur to the same extent in the semitendinosus or biceps femoris muscle. This point is important when developmental findings are later related to carcass or meat quality because a single sampled muscle may not represent whole-body muscular development. Placental insufficiency provides further evidence that fetal growth restriction can affect both fiber size and metabolic identity. Near-term growth-restricted sheep fetuses had smaller hindlimb fibers and lower proportions of type I fibers than normally grown fetuses [31]. Myoblasts isolated from growth-restricted fetuses have also shown impaired proliferative capacity, suggesting that at least part of the response is retained at the cellular level. These findings support a developmental link between poor fetal growth and later muscle metabolism. They do not establish that twins or triplets necessarily develop the same phenotype because the degree of placental insufficiency in experimental models can be more severe than that in an adequately nourished multiple pregnancy.

### 4.4. Programming of Adipose and Connective Tissue Development

Myogenesis occurs alongside adipogenesis and fibrogenesis. These processes share populations of mesenchymal progenitor cells, and their relative activity helps determine the proportions of muscle fibers, adipocytes, and fibroblasts within developing muscle [34]. A shift away from myogenic differentiation may limit muscle formation while increasing the potential for intramuscular fat or connective tissue deposition. Maternal overnutrition and obesity have provided much of the evidence for this developmental competition in sheep. Excess maternal energy intake can suppress Wnt and beta catenin signaling, reduce myogenic differentiation, and promote adipogenic signaling in fetal muscle [35]. Offspring from obese ewes have shown increased lipid accumulation, collagen content, and fibrosis in skeletal muscle, together with impaired insulin signaling [36]. These responses indicate that greater maternal nutrient supply does not invariably improve fetal muscle development. Excess energy can alter the inflammatory and metabolic environment of the fetus and redirect progenitor cell differentiation.

The relevance of these findings to prolificacy requires caution. Prolific ewes are more commonly at risk of insufficient nutrient supply per fetus than maternal obesity induced excess. However, management intended to prevent low birth weight may expose ewes to excessive energy intake if fetal number, body condition, and stage of gestation are not considered. Both inadequate and excessive nutrition may therefore disturb the balance among myogenesis, adipogenesis, and fibrogenesis, although they do so through different physiological conditions. Transcriptomic studies support the sensitivity of fetal muscle and adipose tissue to maternal diet. Changes have been reported in genes involved in tissue development, chromatin regulation, lipid metabolism, and cellular energy use [37]. Maternal diet has also been associated with altered expression and methylation of imprinted genes such as *IGF2R* and *H19* in fetal muscle [38]. These findings provide plausible molecular explanations for persistent developmental effects, but differential gene expression alone does not demonstrate a later change in carcass composition or meat quality.

### 4.5. Persistence of Prenatal Effects After Birth

A prenatal effect is relevant to meat production only if it persists beyond birth or changes the response to postnatal nutrition. Some developmental differences diminish when lambs receive sufficient milk and high-quality diets. Others remain because muscle fiber number, progenitor cell abundance, insulin sensitivity, or adipocyte development has already been altered. Greenwood et al. showed that rapid postnatal nutrition stimulated muscle hypertrophy in lambs of low and high birth weight but did not completely remove differences established before birth [39]. Evidence for persistence beyond the neonatal period is less consistent. Daniel et al. reported that maternal dietary restriction altered muscle fiber composition without markedly reducing final carcass weight [40]. Piaggio et al. found that moderate energy restriction during mid-gestation had limited effects on postnatal growth and feed efficiency after maternal realimentation [41]. Under extensive grazing conditions, prenatal restriction produced effects that depended on lamb sex and the muscle examined, with some differences in metabolic fiber characteristics and meat quality still detectable at slaughter [42].

Available evidence therefore does not support a simple sequence in which larger litter size leads inevitably to poorer muscle development and inferior meat. Placental adaptation, the severity and timing of nutrient limitation, maternal recovery, milk supply, postnatal growth, sex, breed, and slaughter endpoint all influence whether a prenatal difference remains biologically important. The most defensible interpretation is that multiple pregnancy increases the risk of constrained development in susceptible ewes and litters. It does not determine the final phenotype on its own. Representative ovine evidence connecting fetal number, placental development, constrained prenatal growth, and neonatal phenotype is summarized in Table 2. The table distinguishes direct studies of natural litter size from complementary models based on maternal nutrient restriction, low birth weight, or experimentally induced placental insufficiency. In particular, experimental maternal restriction demonstrates the consequences of a defined nutritional insult; its magnitude and timing should not be assigned automatically to naturally occurring twin or triplet gestations.

## 5. Postnatal Growth, Survival, and Carcass Development

Prenatal development establishes much of the biological potential available to the lamb after birth, but it does not determine the final production outcome in isolation. Birth weight, neonatal adaptation, maternal milk supply, rearing environment, health status, and the nutritional conditions experienced after weaning jointly influence how prenatal differences are expressed during growth. Accordingly, the association between litter size and carcass performance often becomes weaker as lambs mature, particularly when animals are managed to a common slaughter weight. This attenuation should not be interpreted as evidence that prenatal effects are unimportant. Rather, it indicates that postnatal management can either reinforce or partially compensate for the disadvantages associated with multiple births.

### 5.1. Birth Weight, Neonatal Viability, and Lamb Survival

Birth weight is one of the most consistent predictors of neonatal survival in sheep. The relationship is generally nonlinear. Lambs at the lower end of the birth weight distribution have limited energy reserves, a large surface area relative to body mass, and reduced capacity to maintain body temperature. Very heavy lambs may also face an increased risk of mortality because of dystocia and prolonged parturition [11,12,13]. The optimum birth weight therefore varies with breed, mature ewe size, sex, litter size, and environmental conditions at lambing [12,27]. Multiple born lambs are more likely to fall below this optimum range because uterine space and placental nutrient supply must be shared among fetuses [26,27,28]. Their lower birth weight is frequently accompanied by delayed standing, weaker teat seeking behavior, and reduced colostrum intake during the first hours after birth [13,14,28]. These limitations are particularly important in triplet and larger litters, in which competition among littermates continues after parturition. Analysis of Australian Merino records showed that mortality to weaning increased from 23 percent in single-born lambs to 32 percent in twins and 45 percent in larger litters [43]. A separate field analysis also indicated that the causes of mortality differed among birth types. Multiple born lambs are more frequently affected by starvation, exposure, mismothering, and complications associated with low birth weight, whereas heavy single-born lambs are relatively more vulnerable to dystocia [44].

Litter size is therefore best considered together with birth weight rather than treated as an independent biological cause of mortality. Within a given litter size, lambs born at an appropriate weight and displaying vigorous neonatal behavior may have survival probabilities comparable with those of single-born contemporaries. Conversely, the survival advantage of singletons can be lost when excessive fetal growth increases the difficulty of birth. Ewe parity, body condition, pelvic dimensions, maternal behavior, lambing supervision, and weather conditions further modify these relationships [12,13,14,43,44]. Triplet production presents the clearest example of the balance required between prolificacy and lamb viability. Reviews of triplet-bearing ewes consistently identify reduced birth weight, lower colostrum intake, impaired thermoregulation, and greater mortality as the main constraints on biological efficiency [45]. Nevertheless, high triplet survival can be achieved when ewe nutrition, shelter, lambing assistance, colostrum management, and litter allocation are coordinated. The number of lambs born is therefore less informative than the number of viable lambs that can be reared under the available production conditions.

### 5.2. Milk Supply, Rearing Type, and Preweaning Growth

During early lactation, milk intake is the principal nutritional determinant of lamb growth. Ewes carrying twins or triplets generally produce more total milk than ewes rearing a singleton, but the increase is not proportional to the number of lambs. Milk available per lamb consequently declines as the rearing group becomes larger. This dilution of maternal resources explains why rearing type often has a stronger effect on preweaning gain than birth type itself [10]. The distinction between birth type and rearing type is essential. A triplet-born lamb reared as a twin may experience a more favorable milk supply than a twin-born lamb reared in an intact triplet group. Cross-fostering, removal of one littermate, artificial rearing, and lamb mortality can therefore alter the postnatal environment without changing prenatal history. Failure to separate these effects may incorrectly attribute reduced weaning weight to fetal development when it is largely caused by postnatal competition for milk.

Data from prolific Polypay sheep illustrate this point. Ewes allowed to rear three lambs produced more total lamb weight than those rearing two, although individual triplet lambs had lower survival and growth rates [46]. Thus, increasing the number reared may improve ewe output while reducing the performance of each lamb. The appropriate rearing strategy depends on milk production, feed availability, ewe condition, labor, and the value placed on uniform slaughter groups. Artificial rearing and fostering can preserve lamb survival when maternal milk is insufficient, but their effects depend greatly on execution. Lambs moved to milk replacer systems may grow more slowly during the initial adaptation period, particularly when the transition occurs abruptly or solid feed intake remains low. With suitable milk replacer composition, hygienic feeding, early access to concentrate, and effective weaning management, much of this difference may disappear later in life. Experimental comparisons indicate that fostering generally maintains growth and carcass performance more consistently than artificial rearing, although both approaches can produce acceptable carcasses under well-managed conditions [47]. Artificial rearing should therefore be viewed as a targeted intervention for surplus lambs rather than a universal solution to high litter size.

### 5.3. Postweaning Compensatory Growth

Differences established before weaning do not necessarily persist unchanged until slaughter. Smaller lambs may exhibit compensatory growth when nutritional constraints are removed, particularly if restriction was moderate and of limited duration. Increased voluntary feed intake and improved efficiency of nutrient use can accelerate weight gain during realimentation. In practical systems, this response may allow multiple-born lambs to narrow the live weight difference from singletons [39,41]. The degree of compensation is variable. It is influenced by age at restriction, restriction severity, duration of realimentation, genotype, sex, health status, and diet quality. Lambs exposed to moderate postweaning restriction may achieve partial or occasionally complete compensation, whereas prolonged restriction during early development can leave persistent effects on frame size, muscle growth, and age at slaughter [40,41,42]. A study of single and twin-born lambs under contrasting growth allowances showed that improved feeding after restriction increased growth rate, but did not guarantee complete convergence among groups [48]. Earlier comparative slaughter work also demonstrated that rapid live weight gain during realimentation may include changes in visceral mass and fat deposition and should not automatically be interpreted as equivalent recovery of saleable muscle [49].

Compensatory growth is therefore better regarded as a conditional biological response than as a predictable correction for low birth or weaning weight. Its production value depends on whether lambs reach the desired carcass specification within an economically acceptable period. Extending the finishing phase may increase feed use, labor, and exposure to health risks even when final carcass weight is recovered.

### 5.4. Slaughter Endpoints, Carcass Yield, and Tissue Distribution

Interpretation of litter size effects on carcass traits depends strongly on the slaughter endpoint. When lambs are slaughtered at a common age, single-born animals usually have greater live weight and carcass weight because of their growth advantage. When slaughter occurs at a common live weight, differences in carcass weight and conformation often become smaller, although multiple-born lambs may require additional days and feed to reach the target [15,16]. Kuchtík et al. reported lower preslaughter and carcass weights in light lambs from larger litters, although the differences were attenuated after adjustment for slaughter weight [50]. The distinction matters because unadjusted comparisons describe the combined consequences of litter size and growth rate, whereas adjusted comparisons describe conditional differences at the specified covariate values. Their causal interpretation depends on the biological role of each covariate. A common carcass-weight endpoint addresses composition at similar carcass size, whereas a common fatness endpoint addresses tissue distribution at similar finishing state. These comparisons do not estimate the same effect: equal live weight may require different ages, and adjustment for carcass weight may remove part of the birth-type association mediated through growth. Statistical adjustment is therefore not causally neutral. Carcass weight may be a mediator along the pathway from birth type through growth to carcass and meat traits, rather than a pre-existing confounder. Adjusting for it can remove part of the association of interest and should not be treated as a universally appropriate correction. Covariate selection should follow the target question, with unadjusted and conditional estimates distinguished explicitly.

Dressing percentage may also be affected by gut fill, fleece weight, organ mass, diet, fasting duration, and maturity. It should not be interpreted as a direct measure of muscularity. Likewise, carcass conformation and fat score reflect both biological development and the slaughter decision. Delaying slaughter until smaller multiple-born lambs reach the same weight as singletons may improve conformation but can also increase fatness. Conversely, slaughter at a fixed age can produce lighter and less uniform carcasses from large litters. Tissue distribution provides a more informative biological endpoint than carcass weight alone. Prenatal and early postnatal restriction can reduce absolute muscle mass, but differences in muscle proportion may be modest once carcass weight is accounted for [39,40,41,42]. Fat deposition is more responsive to the nutritional environment during finishing and to physiological maturity. Accordingly, litter size effects on carcass fatness are often inconsistent among studies because they are confounded by diet, age, weight, sex, and production system. McHugh et al. found a strong genetic association between postweaning live weight and carcass weight, whereas birth weight was a relatively weak predictor of age at slaughter and carcass performance [51]. This supports the use of repeated growth records rather than reliance on birth weight alone when evaluating lambs from prolific flocks.

### 5.5. Individual Lamb Performance Versus Ewe Level Carcass Output

The central production question is not whether a twin or triplet lamb is heavier than a singleton. It is whether a ewe producing a larger litter delivers more marketable carcass weight without an unacceptable increase in mortality, feed demand, labor, or carcass variability. Individual lamb weight generally declines as litter size increases, but total litter weight at weaning and total carcass output per ewe may still rise [3,4,8,46]. This distinction changes the interpretation of prolificacy. A system based solely on individual growth rate will tend to favor singletons, whereas a system evaluated on kilograms of acceptable carcass produced per ewe may favor twins or carefully managed triplets. The advantage diminishes when additional lambs have poor survival, require artificial rearing, remain substantially longer in the finishing system, or fail to meet carcass specifications. Bioeconomic modeling indicates that the optimum flock litter size is therefore system dependent rather than biologically fixed [4].

Evaluation at ewe-level should include the number of lambs slaughtered, total carcass weight, days to slaughter, carcass specification, supplementary feed use, replacement requirements, and ewe reproductive performance in subsequent seasons. These outcomes connect prolificacy with commercial meat production more directly than litter size or individual lamb weight considered separately. They also provide the necessary transition from growth and carcass development to the evaluation of meat quality.

## 6. Litter Size and Lamb Meat Quality

The relationship between litter size and lamb meat quality is less consistent than its relationship with birth weight, growth, and carcass weight. Single born lambs usually grow faster and reach commercial slaughter targets earlier than multiple-born lambs. However, many differences in meat quality become smaller when lambs are compared at a similar carcass weight, physiological maturity, and postmortem handling conditions. A broad assessment of sheep meat production confirms that animal age, slaughter weight, diet, sex, genotype, preslaughter stress, carcass chilling, and ageing often explain more variation in meat quality than birth type [52]. Hanoğlu Oral et al. observed stronger birth type effects on carcass weight and muscularity than on the physicochemical properties of meat [53]. Litter size may nevertheless influence meat quality through its effects on prenatal muscle development, birth weight, maternal milk supply, postnatal growth, finishing duration, and age at slaughter. Its contribution is therefore more likely to arise through an accumulation of indirect effects than through a single persistent pathway. Distinguishing these pathways is essential when evaluating whether increased prolificacy compromises the quality of the resulting lamb meat. The limited number of adequately separated triplet comparisons and differences in slaughter endpoint preclude a universal null effect. The evidence should be synthesized by comparison endpoint. At the same chronological age, contrasts describe developmental outcomes at a given time, including differences in growth, carcass size, and fatness. At the same live weight, they incorporate differences in slaughter age, finishing duration, and compensatory growth, while dressing percentage and carcass weight may still vary. At the same carcass weight, contrasts describe meat traits at comparable carcass size, but animals may differ in age and fat cover. At comparable fatness or a defined finishing endpoint, contrasts address quality at a similar finishing state, while weight, age, and feed use may differ. Consequently, attenuation of a difference at one endpoint does not establish its absence at another or exclude an effect mediated through growth [15,16,39,40,41,42,50,53].

### 6.1. Postmortem Metabolism, pH, and Water Holding Capacity

The conversion of muscle to meat depends on the amount of glycogen present at slaughter and the subsequent production of lactic acid. The extent and rate of pH decline affect protein denaturation, sarcomere shortening, water distribution, proteolysis, meat color, and microbial stability. Animal related factors influence this process, but conditions immediately before and after slaughter are often decisive [54]. Genetic background, nutrition, physical activity, stress, and carcass processing can interact in determining the final meat phenotype [55]. There is currently little evidence that litter size exerts a consistent independent effect on ultimate pH. Multiple born lambs may have different growth trajectories and finishing periods, but these differences do not necessarily result in altered muscle glycogen at slaughter. Glycogen reserves are more directly affected by recent feed intake, health status, physical activity, transport, fasting, handling, and the duration of preslaughter stress. A large analysis of lamb color variation showed that production factors influenced fresh meat color more strongly than myoglobin concentration and pH alone, illustrating the difficulty of assigning a postmortem outcome to one early life factor [56].

The slaughter endpoint is particularly important. When lambs are slaughtered at a common age, multiple-born animals are often lighter and have less carcass fat. Reduced insulation may increase the rate of carcass cooling and the risk of cold shortening. When slaughter occurs at a common weight, multiple-born lambs may be older and may have experienced a longer finishing period. These designs address different biological questions and can produce apparently conflicting results. Water holding capacity is governed by ultimate pH, the rate of early pH decline, carcass temperature, sarcomere length, muscle structure, and postmortem proteolysis. De Brito et al. showed that marked differences in forage type and growth did not produce corresponding changes in drip loss or cooking loss [57]. Comparisons between grass and concentrate systems have similarly demonstrated that feeding strategy can affect carcass composition and selected quality attributes without producing a uniform response in water retention [58]. Antioxidant supply and packaging conditions further influence protein and lipid oxidation during storage. Vitamin E supplementation can delay discoloration and lipid oxidation in lamb meat displayed under a modified atmosphere [59]. Similar benefits have been reported when vitamin E was incorporated into lamb diets before slaughter [60]. Packaging type and the duration of chilled storage can also alter purge loss, color stability, and oxidative deterioration independently of birth type [61]. Studies that have directly or indirectly considered litter size have usually reported limited differences in cooking loss and water-holding capacity [16,41,53]. Where differences occur, they are difficult to separate from carcass weight, fat cover, slaughter age, and chilling rate. Preslaughter transport represents another important source of variation because transport conditions can modify stress responses, muscle energy reserves, and subsequent meat quality [62]. Litter size should therefore not be described as a direct determinant of pH or water-holding capacity unless slaughter conditions and carcass characteristics have been adequately controlled.

### 6.2. Tenderness, Muscle Structure, and Eating Quality

Tenderness is influenced by sarcomere length, connective tissue content and solubility, muscle fiber characteristics, intramuscular fat, calpain activity, carcass chilling, electrical stimulation, and ageing. Animal age is also important because collagen crosslinking generally increases with maturity. Analysis of meat from lambs and older sheep showed that sensory tenderness, juiciness, flavor, and overall liking declined as age and shear force increased and improved as intramuscular fat increased [63]. The relationship between litter size and tenderness is biologically plausible but not straightforward. Primary and secondary muscle fibers develop before birth, and the total number of fibers is largely established during fetal life. Reduced nutrient availability in large litters may limit secondary fiber formation and alter subsequent muscle hypertrophy. Nevertheless, a lower prenatal growth rate need not produce tougher meat. Postnatal nutrition can increase fiber size, while ageing and proteolysis can moderate structural differences present at slaughter.

Consumer-based grading research has identified intramuscular fat, shear force, animal age, sex, and cut as relevant predictors of eating quality [64]. Intramuscular fat is particularly important because it contributes to juiciness and flavor and can reduce the perception of toughness. Pannier et al. reported positive associations between intramuscular fat and sensory acceptance in Australian lamb, with the magnitude varying among muscles and consumer groups [65]. Selection for greater lean meat yield may reduce intramuscular fat and thereby create a tradeoff between carcass yield and eating quality. Genotype and muscularity can further modify tenderness. Lambs selected for greater muscularity do not always produce less acceptable meat, but extreme muscle development may be accompanied by lower intramuscular fat and altered proteolytic characteristics [66]. Breed, sex, maturity, and nutritional management may contribute to eating quality, although their effects are difficult to separate when several production factors change simultaneously [67]. Comparisons among protected-origin lamb breeds have also shown that live weight and breed can affect tenderness and flavor intensity [68]. Wider comparisons among lamb breed types confirm that sensory differences occur, but substantial variation remains within each genotype [69].

Direct comparisons of single and multiple-born lambs seldom demonstrate a consistent difference in shear force [16,41,53]. Single born lambs may appear more tender when they are slaughtered younger at a given weight, while multiple-born lambs may have similar tenderness when carcass weight, fatness, chilling, and ageing are standardized. Conversely, slaughtering all lambs at the same age can result in lighter and leaner multiple-born carcasses that cool more rapidly. The observed response may then reflect carcass chilling rather than prenatal litter size. The current evidence therefore supports a cautious interpretation. Prenatal growth can affect muscle structure, but instrumental or sensory tenderness is the final outcome of several processes acting before and after slaughter. Evidence for developmental changes in muscle is stronger than evidence for an unavoidable tenderness penalty in multiple-born lambs. Reports of birth-type differences should therefore specify whether age, live weight, carcass weight, fatness, and processing conditions were matched or included as covariates; residual differences under one endpoint cannot be extrapolated to another.

### 6.3. Meat Color and Oxidative Stability

Fresh lamb color depends on myoglobin concentration, myoglobin oxidation, muscle pH, oxygen consumption, mitochondrial activity, surface light scattering, lipid oxidation, and retail display conditions. Physical activity and age tend to increase myoglobin concentration, whereas diet affects antioxidant deposition and the susceptibility of membrane lipids to oxidation [52,54]. Hanoğlu Oral et al. reported greater redness and color saturation in single-born lambs than in multiple-born lambs [53]. These findings cannot readily be generalized because birth type is frequently associated with slaughter weight, age, carcass fatness, and growth rate. Multiple born lambs slaughtered at the same live weight may be older and could therefore have higher myoglobin concentrations. Lambs slaughtered at a common age may be lighter and have lower fat cover, which can alter chilling and surface color. The direction of the association may therefore change according to the experimental design.

Diet and production system have clearer effects on color stability. Pasture feeding often produces darker and redder meat because of increased physical activity and higher myoglobin concentration, although responses vary with forage quality and lamb growth rate [57,58]. Vitamin E deposited in muscle protects cell membranes and myoglobin from oxidation. Supplementation can delay discoloration and lipid oxidation during retail display, particularly when meat is stored in oxygen-rich packaging [59,60]. Packaging type and storage duration can themselves alter color stability to an extent that exceeds many animal related effects [61]. Oxidative stability is also influenced by fatty acid composition. Increasing the proportion of polyunsaturated fatty acids can enhance the nutritional value of lamb meat but may increase its susceptibility to lipid oxidation. The balance between polyunsaturated fatty acids and tissue antioxidants is therefore more informative than either component considered alone. There is currently insufficient evidence that litter size systematically alters this balance. Any apparent association is more likely to reflect differences in milk intake, forage exposure, concentrate feeding, fatness, or finishing duration.

### 6.4. Chemical Composition, Fatty Acids, Flavor, and Consumer Acceptance

The chemical composition of lamb meat changes with physiological maturity. Protein concentration is comparatively stable, while moisture declines as intramuscular fat accumulates. Single born lambs may contain more intramuscular fat when slaughtered at a common age because they have grown faster and reached a more advanced stage of maturity. This difference often decreases when animals are compared at a common carcass weight or degree of fatness. Flavor is one of the strongest drivers of consumer acceptance of sheep meat, followed by tenderness and juiciness [70]. Characteristic sheep meat flavor arises from lipid-derived compounds, branched-chain fatty acids, sulfur containing compounds, and reactions involving water-soluble precursors during cooking. Consumer acceptance also depends on cultural familiarity with sheep meat. Consumers from different countries can respond differently to the same flavor compounds [71]. Origin and grazing system can influence purchasing expectations and sensory evaluation, but their importance varies between consumer populations [72]. The interaction between sustainable production, product identity, and consumer preference further demonstrates that eating quality cannot be evaluated from instrumental measurements alone [73].

Comparisons among contrasting lamb production systems have shown that meat from pasture and concentrate systems can differ in fatty acid composition, flavor intensity, and consumer preference. Lambs derived from different breed and production systems can show distinct muscle lipid profiles even when differences in basic eating quality remain relatively small [74]. Diet also influences fat deposition and the balance among saturated, monounsaturated, and polyunsaturated fatty acids [75].

Rumen biohydrogenation considerably modifies the relationship between dietary fatty acids and those deposited in muscle. Botanical composition and forage quality influence ruminal fatty acid metabolism and the fatty acid composition of muscle and subcutaneous fat [76]. Maternal diet may also contribute. Linseed supplementation during pregnancy and lactation has been shown to alter the intramuscular fatty acid composition of suckling lambs, demonstrating that early nutritional exposure can influence meat lipids before conventional finishing begins [77]. Pasture and legume based systems can improve the proportion of nutritionally desirable fatty acids, although their effects on oxidative stability vary. Sainfoin supplementation has been associated with changes in muscle fatty acid composition and lipid stability, partly because condensed tannins can modify ruminal lipid metabolism and antioxidant status [78]. Supplementation with antioxidant rich ingredients can further protect polyunsaturated lipids during storage. Red wine extract and vitamin E supplied in combination with linseed and fish oil have been evaluated as means of maintaining meat quality while improving the lipid profile [79]. Rosemary diterpenes can also delay the formation of rancid volatile compounds during storage under a protective atmosphere [80].

Production systems may alter flavor through the formation of skatole, indole, and other volatile compounds. Comparisons between organic and conventional pasture systems have revealed differences in nutritional composition and selected flavor-related traits even when lambs were managed under generally comparable grazing conditions [81]. Fresh alfalfa supplementation can modify skatole and indole concentrations in fat and can influence chop odor and flavor [82]. The proportion of pasture and concentrate in the finishing diet also affects sensory responses. Increasing concentrate intake before slaughter may reduce some pastoral odor and flavor characteristics [83]. The duration of finishing is relevant because short periods of concentrate feeding may alter the volatile profile without fully removing compounds accumulated during grazing. Gkarane et al. found that finishing diet and its duration jointly influenced volatile composition and sensory quality [84]. These effects are particularly relevant to prolific systems. Multiple born lambs commonly require additional time or supplementary feeding to reach a target carcass weight. Their meat may therefore differ from that of singletons because of the finishing strategy used to compensate for slower early growth. Such differences should be attributed to the complete production pathway rather than to litter size alone. Direct sensory comparisons among single, twin, and triplet lambs remain too limited to conclude that consumers systematically prefer meat from one birth type.

### 6.5. Prenatal Programming and Persistence of Meat Quality Effects

The biological argument linking litter size with meat quality rests largely on fetal programming. Natural increases in litter size can reduce placental nutrient availability and restrict fetal growth [26,27]. Controlled maternal restriction studies provide additional evidence that prenatal nutrition affects muscle development, although these models do not fully reproduce the heterogeneous conditions of twin and triplet pregnancy. Maternal undernutrition has produced sex-dependent effects on lamb growth, carcass characteristics, and meat quality [85]. Poor maternal nutrition can reduce fetal muscle growth, alter myogenic progenitor populations, and modify the expression of genes involved in cell proliferation, protein synthesis, and energy metabolism [33]. Late gestational nutrient restriction has also been associated with changes in muscle and bone development that remain detectable several months after birth [86]. Fetal muscle, adipose tissue, and connective tissue originate from related mesenchymal progenitor populations. Nutritional conditions during fetal life may influence the allocation and differentiation of these cells and consequently alter muscle fiber formation, adipogenesis, and connective tissue deposition [87]. The resulting phenotype depends on the timing and severity of restriction and on nutrient supply after birth. Postnatal growth can either magnify or moderate prenatal effects. Genetic potential and nutrient supply jointly regulate muscle growth and muscle fiber characteristics [88]. Lambs subjected to different nutritional histories may exhibit compensatory live weight gain after realimentation, but recovery of live weight need not indicate equivalent recovery of muscle and fat distribution [89]. Hopkins et al. showed that lambs reaching a comparable final weight could differ in carcass composition and production efficiency because of their earlier growth paths [90].

Some prenatal effects may therefore remain biologically detectable without causing a commercially important reduction in meat quality. A measurable difference in muscle fiber size, gene expression, or lipid metabolism does not automatically translate into unacceptable tenderness, flavor, or shelf life. Mortimer et al. estimated variable heritabilities among meat quality traits and identified both favorable and unfavorable correlated responses involving carcass leanness, muscle depth, intramuscular fat, and eating quality [91]. Litter size must consequently be evaluated within the genetic and nutritional background of the flock. The balance of current evidence is summarized in Table 3. Strong and repeatable effects are evident for birth weight, early growth, slaughter age, and carcass weight. Evidence becomes progressively less consistent for intramuscular fat, color, tenderness, and fatty acid composition after slaughter weight, age, diet, and postmortem conditions are taken into account. Taken by endpoint, common-age comparisons are most informative about growth-related differences in carcass readiness and composition. Common-live-weight comparisons must retain the accompanying differences in age and feeding history; they should not be grouped with common-carcass-weight comparisons. Similar carcass weight can reduce differences associated with carcass size without equalizing fatness or physiological maturity. Comparisons at similar fatness further separate finishing state from growth duration. Across these endpoints, evidence for a consistent birth-type effect on pH, water retention, or tenderness remains limited, and the absence of a difference at a matched endpoint does not negate the extra time or resources required to reach it.

### 6.6. Evidence Gaps and Priorities for Future Research

Several limitations prevent stronger conclusions. Many experiments were designed primarily to examine breed, diet, maternal nutrition, or slaughter weight. Birth type was often included only as an adjustment term, and the number of triplet lambs was commonly too small to support a separate analysis. Birth type and rearing type were not always distinguished, even though prenatal competition and postnatal milk availability represent different biological exposures. The use of different slaughter endpoints further complicates comparison among studies. Variation in meat sampling and analysis is also important. Studies differ in muscle selection, sampling time, ageing duration, cooking endpoint, shear force protocol, display conditions, and adjustment for carcass weight or fatness. Rapid methods such as near-infrared spectroscopy may support large-scale evaluation of fatty acid composition, but calibration across breeds and production systems remains necessary [92]. Evidence generated from a limited number of carcasses under one management system should therefore not be generalized to prolific sheep populations without independent validation. Contradictory meat-quality results should be interpreted against the endpoint, balance of breed and diet groups, and whether triplets were analyzed separately; a null finding from a small subgroup is not evidence of equivalence.

Commercial lamb populations raised under different forage systems frequently show substantial overlap in carcass and meat quality despite clear differences in growth and diet [93]. This observation reinforces the need to study litter size within the complete production system. Future experiments should follow identified lambs from pregnancy to meat evaluation, distinguish the number born from the number reared, and record colostrum intake, milk access, growth path, health treatments, and finishing diet. Single, twin, and triplet lambs should be evaluated at both a common age and a common carcass endpoint. Measurements should include muscle glycogen, pH and temperature decline, water-holding capacity, intramuscular fat, muscle fiber characteristics, connective tissue properties, proteolysis, oxidative stability, fatty acid composition, volatile compounds, and consumer sensory responses. Candidate model terms include ewe, sire, sex, birth weight, rearing type, diet, slaughter group, carcass weight, and postmortem processing. Their inclusion should follow the sampling or treatment structure and the target effect, rather than a fixed list of covariates. Birth weight and carcass weight may mediate the association under investigation. Ewe and litter should be treated appropriately in the experimental structure because lambs born to the same ewe are not biologically independent. Analyses should specify the estimand and account for lambs nested within litters and ewes, with appropriate sire, flock, year, and management-group effects where the sampling design supports them. Mixed-effects or multilevel models should address shared litter environments, and repeated body weights should be analyzed longitudinally with within-lamb correlation rather than as independent observations at each age. Prespecified contrasts and confidence intervals are especially important for sparse triplet groups. The experimental unit should be defined before analysis from the level at which treatment is assigned or exposure is compared; it is not automatically the individual lamb. For observational birth-type comparisons, the lamb may be the measurement unit, while the litter or ewe defines a shared exposure and dependence structure. Treating littermates as independent replicates without accounting for this structure can cause pseudoreplication. Sire, flock, year, and management-group effects, where identifiable, need not all be random effects: their treatment as fixed, random, or stratification factors depends on the number of levels, sampling structure, and inferential objective.

Current evidence does not support the conclusion that prolificacy inevitably reduces lamb meat quality. Its more consistent consequence is increased variation in early growth, finishing duration, slaughter age, and carcass maturity. These differences can create variation in meat quality when lambs are managed as a single group. Appropriate nutritional support, separate drafting, and slaughter according to carcass readiness may preserve product quality while retaining the ewe-level advantage in total lamb and carcass output. A minimum design and reporting framework for separating prenatal litter size effects from postnatal management and slaughter effects is provided in Table 4.

## 7. Integrated Strategies for Balancing Prolificacy and Lamb Meat Production

Increasing litter size can improve the amount of lamb produced per ewe, but the response is not linear. Beyond the biological and managerial capacity of the flock, additional fetuses increase the risks of low birth weight, lamb mortality, delayed growth, variable slaughter age, and greater dependence on supplementary feeding. The practical objective should therefore be an optimum litter size rather than the highest attainable litter size. This optimum will differ among breeds, environments, feed resources, labor structures, and target markets. A balanced strategy requires coordination among maternal and terminal selection, pregnancy nutrition, lambing management, postnatal feeding, and slaughter decisions. None of these measures is sufficient alone. Genetic improvement can increase reproductive and growth potential, but management determines whether that potential is converted into viable lambs and saleable carcasses.

### 7.1. Breeding for an Optimum Rather than Maximum Litter Size

Litter size has relatively low heritability and is influenced by ewe age, parity, nutrition, season, and reproductive management. Genetic progress through direct selection is therefore possible but generally slower than progress for growth or carcass traits. The use of prolific breeds, crossbreeding, and major reproductive genes can accelerate change, although the resulting biological response may exceed the capacity of extensive production systems [94]. Estimates compiled across sheep populations show that reproductive traits usually have lower heritability than live weight, muscle depth, or fat depth [2]. Selection based only on lambs born per ewe may increase prenatal competition and reduce individual birth weight. A more useful objective is the weight of lamb weaned per ewe, which combines prolificacy, maternal ability, survival, and preweaning growth. Genetic relationships among litter weight, prolificacy, growth, and wool traits support the use of ewe output as an integrated selection criterion [95]. French meat sheep data likewise suggest that litter weight can complement litter size and early growth in maternal selection programs [96]. Evidence from commercial flocks supports the use of balanced maternal and terminal indexes. Ewes with superior maternal index values have produced larger litters, improved perinatal survival, heavier lambs, and fewer days to slaughter [97]. Economic breeding objectives should therefore assign appropriate weights to litter size, lamb survival, mature ewe weight, feed demand, growth rate, carcass weight, conformation, and fatness. These weights should reflect the production environment and payment system rather than remain fixed across countries or breeds [98].

Selection for maternal performance also needs to account for ewe maintenance requirements. Larger and more productive ewes may wean heavier litters, but their annual feed demand is greater. In low-input hill systems, breeding objectives that incorporate ewe longevity, maternal ability, lamb survival, and mature size can offer a more realistic measure of efficiency than output per lamb [99]. Across country genetic evaluation may further increase the accuracy of breeding values for growth and carcass traits when populations share breeds and recording systems [100]. Lamb survival should be treated as a breeding objective in its own right. Survival is genetically complex and is influenced by direct lamb effects and maternal effects. Riggio et al. identified genetic variation in neonatal survival, although the estimated heritability was low [101]. Survival recorded at birth, during the neonatal period, and to weaning may represent partially different traits because the predominant causes of mortality change over time [102]. Genetic improvement is therefore more likely when survival records are collected at clearly defined ages and combined with birth weight, lambing ease, and maternal behavior [103]. Hinch and Brien identified birth weight, litter size, dystocia, starvation, exposure, mismothering, and weather conditions as major determinants of lamb mortality [104]. Genetic evaluation cannot remove the need for suitable lambing management, but it can gradually shift the population toward improved vigor, maternal care, and survival. Reproductive traits should also be evaluated across repeated parities because ewe performance in one lambing does not fully describe lifetime productivity [105].

Major genes affecting ovulation rate offer opportunities and risks. The Booroola mutation in the receptor gene *BMPR1B* can markedly increase ovulation rate and litter size [5]. Mutations in *BMP15* and *GDF9* can produce similar effects, although homozygous and heterozygous animals may differ substantially in fertility [7,17,18]. Mutations involving genes in related ovarian pathways have also been associated with increased ovulation rate in prolific breeds [6]. Marker assisted selection should therefore consider genotype dosage, breed background, expected litter size distribution, and management capacity. Genomic information is increasingly being used to identify pathways associated with ovulation, embryo survival, maternal performance, growth, and carcass composition [106]. Reviews of candidate genes affecting prolificacy show that reproductive variation is distributed across several interacting pathways rather than controlled by a single universal marker [107]. Accordingly, genomic selection based on large and accurately phenotyped populations is likely to be more reliable than selection based on isolated candidate polymorphisms [108]. Recent estimates also confirm that increasing genetic merit for litter size can reduce individual lamb weight, reinforcing the need to include birth and weaning performance in the same breeding objective [109]. Improved ovulation does not guarantee more lambs reared: allele dosage can alter fertility, the litter-size distribution, neonatal survival, maternal demand, and the frequency of assisted rearing. The relative value of candidate-marker and genome-wide approaches must therefore be established within the breed and recording population rather than assumed for every flock.

### 7.2. Pregnancy Diagnosis and Nutrition According to Fetal Number

Ultrasonographic pregnancy diagnosis provides the first practical opportunity to align feed supply with fetal number. Separating barren, single-bearing, twin-bearing, and triplet-bearing ewes allows nutrients to be directed toward animals with different requirements. Late gestational nutrition is especially important because fetal growth accelerates while rumen capacity is constrained by the gravid uterus. Nutritional management during this period influences birth weight, colostrum production, ewe condition, and subsequent milk supply [110]. For single-bearing ewes, the feed allowance should reflect body condition score (BCS), mature size, forage supply, and gestational stage; excessive energy intake in an already well-conditioned ewe may increase feed costs and, in some circumstances, dystocia risk. Twin bearing ewes generally require greater dietary energy and metabolizable protein, while triplet-bearing ewes face the greatest risk of negative energy balance, pregnancy toxemia, low birth weight, and inadequate colostrum production. Nutritional responses also depend on ewe size, body condition, parity, forage quality, and lambing season [45]. Thompson et al. found that survival in triplet-bearing ewes and their lambs varied with breed, ewe condition, fetal burden, and management between pregnancy scanning and lamb marking [111]. Increasing concentrate supply during late pregnancy can reduce ewe mortality in some circumstances, although greater supplementation does not invariably improve triplet lamb survival [112]. Managing ewe body condition between scanning and lambing appears more consistent than relying on short periods of intensive feeding immediately before birth [113]. Fetal count should therefore guide grouping rather than prescribe a fixed concentrate allowance. The reported reduction in ewe mortality with supplementation applies to the studied nutritional and management conditions and should not be generalized to adequately fed flocks [112]. Triplet-bearing ewes also vary in parity, condition, breed, and capacity to consume supplementary feed. BCS is an operational measure for nutritional planning, rather than a general description of ewe condition. It should be assessed on a consistent, recorded scoring scale at mating, scanning, before lambing, and during lactation, and interpreted alongside live weight and fetal count. Requirements and supplementation responses also depend on production potential, mature size, parity, feed availability and quality, and gestational stage. Ewes with the same fetal number may therefore need different feeding strategies; BCS targets and supplementation should be set for the breed and production conditions rather than inferred from fetal count alone [45,110,111,112,113,114].

Commercial farmers who achieve relatively high triplet survival commonly combine pregnancy scanning, separate nutritional management, smaller lambing groups, accessible shelter, frequent observation, and rapid colostrum intervention [114]. These practices are complementary. Increasing dietary allowance without addressing lambing density, exposure, or delayed suckling is unlikely to produce the full expected benefit. Nutritional management before and around conception may also influence placental development and subsequent lamb vigor. Periconceptional restriction can alter fetal growth, neonatal behavior, and survival even when maternal nutrition improves later in pregnancy [115]. This observation argues against restricting management attention to the final weeks of gestation. Ewe condition should be monitored from mating through lactation. In intensive systems, additional labor, individual observation, and artificial rearing may be feasible; in extensive pasture systems, stocking pattern, shelter, available feed, and realistic intervention capacity determine which measures can be implemented [45,114].

### 7.3. Lambing Management, Colostrum, and Rearing Allocation

Most lamb mortality occurs during the first few days after birth. Low birth weight, dystocia, hypothermia, starvation, and mismothering frequently occur together rather than as isolated causes [116]. Management should therefore focus on the short period between the onset of parturition and the establishment of a stable ewe and lamb bond. Lamb vigor is affected by prenatal growth, birth difficulty, sex, litter size, and maternal nutrition. Multiple born lambs tend to stand and suck later than singletons and have less time to obtain an adequate share of colostrum [117]. Maternal undernutrition can further delay standing and teat seeking behavior and can reduce the ability of lambs to maintain body temperature [28]. Colostrum provides energy, immunoglobulins, water, fat soluble vitamins, and growth related signals. Its volume and viscosity are influenced by ewe nutrition and hormonal changes before parturition. Improved nutritional supply during late pregnancy can increase colostrum production and reduce the risk that multiple-born lambs receive an inadequate amount [118]. Short term supplementation can be beneficial when pasture quality is insufficient, although responses vary among breeds and feeding systems [119]. The ewe and lamb bond also influences whether available colostrum is successfully consumed. Early maternal grooming, low disturbance, rapid lamb recognition, and continued proximity promote successful suckling [120]. Competition among littermates can interrupt this process, particularly when the ewe has only two functional teats. Behavioral interactions between the ewe and each lamb are therefore best considered when deciding whether triplets should remain as an intact litter [121]. These are population-level tendencies; birth weight, dystocia, sex, maternal behavior, and exposure may explain much of the variation in time to stand and suck within each birth type [11,12,13,14,28].

Simple maternal behavior scores may assist the identification of ewes that remain close to their lambs and provide attentive care [122]. However, scores collected during routine management can be influenced by human disturbance, parity, breed, and previous experience. Maternal behavior should be combined with lamb vigor and survival records rather than used as a single selection measure. Triplet litters require an explicit rearing decision. Leaving all three lambs with the ewe can maximize litter output when milk production is high and pasture is abundant. Cross-fostering may provide a better biological environment when suitable single-bearing ewes are available. Artificial rearing can protect surplus lambs but increases labor, milk replacer costs, and the risk of digestive and infectious disease. The optimum choice depends on ewe milk capacity, lamb birth weight, litter uniformity, available labor, and the value of the lamb [123].

Thermal support and rapid energy provision are particularly important for small lambs. Brown adipose tissue thermogenesis and early colostrum intake provide the main defenses against hypothermia [124]. Low-birth-weight lambs may require drying, warming, assisted suckling, or measured colostrum administration. Colostrum source and timing should be recorded because the opportunity for timely colostrum intake diminishes when suckling is delayed [120]. Maternal care remains preferable when it is adequate, but intervention should not be postponed in a weak lamb merely to preserve natural rearing. Variation among ewes in maternal behavior, milk production, and udder function means that litter size alone cannot determine rearing capacity [125,126]. A ewe with functional teats, strong maternal behavior, good condition, and high milk yield may rear triplets successfully, whereas another ewe may struggle with twins. Rearing allocation should therefore be based on ewe and lamb phenotype rather than a rigid birth type rule. The immediate decision to assist should follow the lamb’s temperature, vigor, suckling, and colostrum status together with the ewe’s behavior and udder function, irrespective of the recorded birth type.

### 7.4. Postnatal Nutrition, Grouping, and Slaughter Management

Milk production is a major determinant of preweaning growth in multiple-born lambs. Ewes rearing larger litters usually produce more total milk, but milk yield does not increase in direct proportion to lamb number. The amount available to each lamb consequently declines [127]. Creep feeding can reduce this limitation by encouraging early solid feed intake, although its economic value depends on milk supply, forage quality, feed price, and expected slaughter age. Artificially reared lambs require careful control of milk concentration, feeding frequency, hygiene, weaning age, and starter intake. Abrupt restriction of milk before sufficient solid feed consumption is established can delay growth and increase variation within the slaughter group [128]. Where possible, foster rearing or restricted suckling may retain some advantages of maternal care while reducing the milk burden on highly prolific ewes. Suckling duration also affects growth and carcass development. Earlier weaning can allow ewes to recover body condition, but lambs require adequate concentrate and forage intake to avoid a prolonged growth check [129]. Decisions should be based on lamb weight and solid feed intake rather than chronological age alone.

After weaning, multiple-born lambs should not automatically be managed as a uniform group with heavier singletons. Separate drafting by live weight, growth potential, health status, and target market allows dietary energy and protein to be matched more closely to requirements. Birth weight can continue to influence growth and carcass development, even when lambs are slaughtered at a common live weight [130]. However, much of the early disadvantage can be reduced through appropriate postnatal nutrition. Compensatory growth may help smaller lambs approach a target weight, but rapid live weight recovery does not always represent proportional muscle deposition. The response depends on the severity and duration of restriction, physiological maturity, diet quality, and the time available before slaughter [48,49]. Prolonged finishing can increase feed intake, labor, parasite exposure, and the risk of excessive fat deposition. Recovery of live weight must be assessed separately from recovery of muscle-to-fat ratio, fat distribution, and carcass compliance, since these endpoints can follow different trajectories [48,49].

Electronic identification and repeated live-weight recording can improve individual monitoring. Precision livestock technologies offer opportunities to detect changes in feeding behavior, activity, body weight, and health in sheep systems [131]. Their value lies in supporting timely decisions rather than replacing stockmanship. Automated data are most useful when they are connected to pregnancy scan results, birth type, rearing type, growth records, and carcass feedback. Ultrasound measurements of muscle and fat depth can support selection and drafting before slaughter. Genetic parameters for live weight, ultrasound muscle depth, fat depth, and reproductive traits indicate that these characteristics can respond to selection [132]. Brito et al. confirmed exploitable genetic variation in growth, carcass, and meat quality traits in New Zealand sheep [133]. These measurements may help identify multiple-born lambs that have reached an acceptable carcass endpoint despite lower live weight or slower early growth. Carcass feedback should be returned to breeders and producers. Genetic relationships among growth, carcass composition, intramuscular fat, and eating quality mean that selection for one endpoint can change another [91]. Combining live animal records with abattoir data can improve selection accuracy and allow finishing strategies to be evaluated against saleable carcass yield rather than live weight alone [134]. For prolific flocks, the immediate application is to flag lambs whose growth trajectory predicts delayed slaughter or an unsuitable carcass endpoint, using linked birth, rearing, and weight records.

### 7.5. Economic, Welfare, and Environmental Evaluation

The benefit of increasing litter size should be evaluated per ewe and per unit of feed rather than per surviving lamb alone. Biological indicators include lambs born and reared per ewe, kilograms of lamb weaned and saleable carcass produced per ewe, feed efficiency, ewe survival and longevity, days to slaughter, and carcass compliance. Economic indicators include revenue, feed, labor, veterinary and replacement costs, margin per ewe, and margin per kilogram of saleable carcass. These measures answer different questions. Increased biological output can coexist with a lower margin when additional feed, care, or replacement costs exceed the value of the extra carcass output [3,4]. A prolific system may produce more carcass weight per ewe but still perform poorly if mortality, feed demand, or labor increases excessively [3,4]. Animal welfare is integral to this evaluation. Large litters are associated with a greater risk of low birth weight, starvation, hypothermia, and mismothering. Triplet bearing ewes may also experience greater metabolic stress and mortality [45,111,112,113,114]. These outcomes cannot be treated as acceptable production losses merely because total lamb output remains high. Welfare assessment should include the ewe as well as the lamb. Environmental efficiency is similarly dependent on the denominator used. Greater lamb output can dilute the maintenance requirements and environmental burden of the breeding ewe across more kilograms of meat. This advantage is reduced when additional lambs die or require long finishing periods. Production efficiency should therefore be calculated using saleable carcass weight rather than fetuses conceived or lambs born. Welfare outcomes should be explicit breeding and management criteria, including neonatal morbidity and mortality, the frequency of assisted birth or feeding, ewe health and longevity, and the labor required to provide timely care. Economic return additionally depends on feed and labor prices, carcass payment rules, veterinary expenses, and replacement costs; the preferred litter-size distribution should be estimated for each system [3,4]. Environmental comparisons likewise require a stated system boundary and functional unit: conclusions expressed per ewe, per lamb born, per lamb reared, per unit of live weight, or per kilogram of saleable carcass may differ. The functional unit can change both the magnitude and interpretation of an apparent environmental advantage. Lower emissions or resource use per kilogram of saleable carcass do not necessarily imply lower emissions or resource use per ewe maintained, because output and inputs may rise together. Both denominators should be reported where relevant within the same stated system boundary.

Commercial forage systems demonstrate that different growth pathways can produce broadly acceptable carcasses, although variability in finishing time and product composition remains [93]. The appropriate strategy will differ between intensive flocks with access to concentrate and labor and extensive flocks that depend on pasture and limited supervision. A single recommended litter size therefore cannot be applied across all systems. The principal strategies and their tradeoffs are summarized in Table 5. Each intervention is positioned within the stage when it is most likely to be effective. The table also distinguishes improvement in individual lamb performance from improvement in ewe-level output. Future research needs to combine reproductive, survival, growth, carcass, and meat quality records within the same population. Electronic identification, genomic information, repeated live-weight measurement, ewe condition records, and abattoir feedback provide the components needed for such an approach. These data should be analyzed at both lamb and ewe-levels because improvement in individual lamb performance does not always correspond to the greatest flock output. Priority questions and study designs needed to define biologically and commercially optimum prolificacy are summarized in Table 6. The most useful breeding objective will be one that increases the probability of a ewe producing a manageable litter of vigorous lambs that reach an acceptable carcass endpoint within a predictable period. This approach does not reject prolificacy. It places prolificacy within the biological limits of the ewe, the management capacity of the farm, and the quality requirements of the market. Prospective whole-flock bioeconomic analysis should test alternative litter-size distributions under locally observed prices, resources, and welfare constraints rather than assume a transferable economic optimum. Broad labels such as intensive and extensive do not fully describe management feasibility. Climatic conditions, forage availability and quality, concentrate access, labor, housing and handling infrastructure, veterinary services, and farm financial resources affect the interventions that can be sustained. Two extensive systems may face very different constraints because of their geographical, climatic, and socioeconomic settings. Recommendations should therefore specify the resources and conditions required for implementation.

## 8. Conclusions

Prolificacy can raise lamb output per ewe, yet the gain is realized only when additional fetuses become surviving lambs and marketable carcasses. Increasing fetal number intensifies competition for uterine space and placental supply, producing lower and more variable birth weights. The associated risks involve muscle development, vigor, thermoregulation, colostrum intake, survival, and early growth and are greatest in triplet and larger litters. By contrast, evidence for a uniform deterioration in meat quality is weak. Multiple born lambs commonly take longer to reach slaughter, but differences in ultimate pH, water-holding capacity, color, tenderness, intramuscular fat, fatty acid profile, and sensory acceptance are often small or inconsistent once age, diet, carcass weight, fatness, and postmortem handling are considered. Litter size thus affects the route and time to a commercial endpoint more reliably than the intrinsic quality of the meat. This conclusion applies to the heterogeneous breeds and comparisons reviewed and is conditional on the slaughter endpoint, nutritional history, carcass fatness, and postmortem treatment; triplet-specific evidence remains limited.

Production systems should pursue optimum litter size, not maximum prolificacy. Selection for lambs born alone can increase mortality, ewe nutrient demand, labor, and carcass variation. A defensible breeding objective combines reproduction with survival, maternal ability, mature ewe size, growth, carcass value, and meat quality, and evaluates saleable carcass output per ewe against biological and economic cost. Pregnancy scanning, nutrition matched to fetal number, appropriate ewe condition, prompt colostrum support, deliberate allocation of surplus lambs, targeted postweaning feeding, weight-based grouping, and slaughter according to carcass readiness can reduce losses, particularly in triplet-bearing ewes. These relationships are integrated in Figure 1, which illustrates how maternal prolificacy is translated through fetal number, cotyledonary placental function, prenatal development, neonatal survival, and postnatal growth into carcass and meat-quality outcomes. The framework also identifies stage-specific management opportunities and distinguishes the well-supported consequences of realized litter size from the still uncertain direct effects of prolificacy-associated alleles. The target litter-size distribution must be set for each production system by following lambs born through survival, rearing, and marketing while accounting for ewe health and longevity, intervention frequency, and labor requirements. Sustainable production requires these welfare and management outcomes to be considered alongside reproductive performance, growth, carcass value, and locally relevant financial margins. More lambs born are beneficial only when the flock can support them without compromising ewe or lamb welfare.

Future work should record birth type and rearing type separately and follow identified lambs from gestation to carcass and meat evaluation. Comparisons at a common age and at a common carcass endpoint are both needed because they answer different questions. Linking reproductive, survival, growth, carcass, meat-quality, welfare, and economic records within the same population would allow optimum prolificacy to be estimated rather than assumed. The experimental unit should be defined before analysis. Hierarchical models should account for litter and ewe dependence and for sire, flock, year, and management-group effects where identifiable. Fixed or random treatment should reflect sampling structure, available factor levels, and the inferential objective. Repeated growth measurements require within-lamb correlation. Economic and environmental estimates should state local prices, system boundaries, and functional units. Reporting unadjusted and adjusted estimates helps distinguish the overall birth-type association from conditional comparisons. These contrasts alone do not quantify mediation or establish causality, particularly when growth or carcass weight lie on the pathway under investigation.

## Figures and Tables

**Table 1:** Major genetic determinants of prolificacy in sheep and evidence of their relationships with lamb production traits.

Gene or Locus	Representative Allele or Variant	Representative Breeds or Populations	Principal Reproductive Effect	Inheritance and Fertility Pattern	Evidence Concerning Lamb Growth, Carcass, or Meat Traits	References
*BMPR1B*	FecB	Booroola Merino, Garole, Javanese, Small Tail Han, and Hu sheep	Increased follicular responsiveness, ovulation rate, and litter size	The effect is generally additive, and homozygous carriers usually remain fertile	Associations with lamb performance are mainly mediated through larger litters and altered birth type. Direct effects on muscle development, carcass composition, or meat quality have not been established	[5,7]
*BMP15*	FecXI, FecXH, FecXB, FecXG, FecXL, FecXR, and other breed-specific variants	Inverdale, Hanna, Belclare, Cambridge, Lacaune, Rasa Aragonesa, and other populations	Increased ovulation rate in carriers of several variants	Several loss-of-function variants increase ovulation in heterozygous ewes but cause sterility in homozygous ewes. The response differs among variants	Available studies primarily report reproductive phenotypes. Evidence for direct effects on lamb growth, carcass, or meat quality remains insufficient	[6,17,18]
*GDF9*	FecGH and other FecG variants	Belclare, Cambridge, Thoka, Santa Inês, and other populations	Altered follicular development, ovulation rate, and litter size	Some variants show increased ovulation in heterozygous carriers and infertility in homozygous carriers. Other variants have population-dependent effects	Most evidence concerns ovulation and fertility. Reported offspring differences cannot yet be separated reliably from litter size and maternal effects	[18]
*B4GALNT2*	FecLL	Lacaune and related French populations	Increased ovulation rate through ectopic ovarian expression and altered protein glycosylation	The effect is associated with increased prolificacy without the classical homozygous sterility reported for several BMP15 variants	Direct associations with lamb growth, carcass composition, and meat quality have not been adequately evaluated	[19]
Multiple candidate loci	Population specific single nucleotide polymorphisms and haplotypes	Pelibuey, Polish Mountain Sheep, and other breeds included in genome-wide association studies	Small or moderate associations with litter size, follicular function, or reproductive signaling	Effects are generally polygenic and may not replicate across breeds	Evidence is largely limited to association with reproductive records. Pleiotropic effects on lamb production require independent validation	[20,21]

Gene symbols are italicized, whereas allele and locus designations are presented in standard type. Representative breeds are examples and do not constitute an exhaustive list of all populations in which a variant has been detected. Increased litter size does not demonstrate a direct genetic effect on lamb growth, carcass composition, or meat quality. Here, direct denotes a genotype-attributable effect on lamb growth, carcass composition, or meat quality that is not mediated through litter size. Evaluation must also distinguish maternal genotype, population structure, and rearing environment; a residual genotype association alone does not establish causality. Birth type records lambs delivered together; rearing type records the postnatal group after mortality, fostering, or artificial rearing.

**Table 2:** Representative ovine evidence connecting fetal number, constrained prenatal growth, and neonatal phenotype.

Study Population or Model	Main Comparison	Gestational or Assessment Period	Placental or Fetal Measurements	Principal Findings	Relevance to Litter Size	References
Multiple sheep breeds summarized across published datasets	Singleton, twin, triplet, and quadruplet lambs	Birth	Birth weight and total litter weight	Mean lamb birth weight declined progressively as litter size increased, although total litter weight increased	Provides broad evidence for litter size dependent fetal growth, but breed and management effects cannot be fully separated	[26]
Bluefaced Leicester crossed with Swaledale ewes and Welsh Mountain ewes	Variation in litter size, maternal weight, parity, nutrition, and fetal sex	Throughout gestation and at birth	Maternal energy intake, maternal body composition, litter size, and lamb birth weight	Litter size was the largest measured source of variation in birth weight. Maternal size, nutrition, parity, and fetal sex also contributed	Demonstrates that litter size is important but acts within a wider maternal and environmental context	[27]
Several sheep breeds and parities	Singleton and multiple pregnancies	Placenta collected at parturition	Placental weight, placentome number, lamb birth weight, and neonatal behavior	Placental traits differed with breed, litter size, and parity. Lower placental investment per lamb was associated with lighter lambs and slower neonatal behavioral progress	Directly connects litter size and placental phenotype with neonatal viability	[28]
Placental insufficiency model in pregnant ewes	Growth restricted and normally grown fetuses	Near term	Fetal weight, blood oxygen and glucose, hindlimb muscle fiber size, and fiber type	Growth restricted fetuses were smaller and had smaller muscle fibers with lower proportions of insulin-sensitive type I fibers	Provides mechanistic evidence for the consequences of inadequate placental supply but is not a direct litter size comparison	[31]
Low and high birth weight lambs	Contrasting prenatal growth followed by controlled artificial rearing	Birth to approximately 20 kg live weight	Body composition and skeletal muscle growth	Low birth weight lambs had less muscle at birth and retained differences in muscle development during early postnatal growth, although nutrition modified the magnitude of the response	Shows that consequences associated with restricted prenatal growth can persist independently of postnatal milk supply	[39]
Ewes receiving restricted or adequate diets during pregnancy	Maternal dietary restriction and control feeding	Pregnancy, birth, and later slaughter	Lamb birth weight, muscle fiber composition, carcass characteristics, and muscle development	Maternal restriction altered muscle fiber characteristics, although effects on later carcass traits were not uniform	Supports developmental programming but does not reproduce the full biological complexity of natural multiple pregnancy	[40]

The table includes direct studies of litter size and complementary ovine models of constrained prenatal growth. Findings from maternal nutrient restriction or experimentally induced placental insufficiency should not be interpreted as direct evidence that multiple birth produces the same degree or type of fetal restriction. Birth weight is treated as an indicator of prenatal growth rather than a complete measure of placental function. Differences in breed, fetal sex, maternal age, parity, nutritional treatment, gestational stage, and sampled tissue should be considered when studies are compared.

**Table 3:** Evidence linking litter size and associated developmental conditions with lamb meat quality.

Quality Domain	Main Indicators	General Response Associated with Multiple Birth or Reduced Prenatal Growth	Important Modifying Factors	Evidence for an Independent Litter Size Effect	References
Postmortem metabolism	Muscle glycogen, pH decline, ultimate pH	Usually unchanged, with occasional study specific differences	Stress, fasting, transport, activity, diet, and chilling	Weak	[16,41,52,53,54,62]
Water holding capacity	Drip loss, cooking loss, and purge loss	Most studies report little or no consistent difference	Ultimate pH, carcass temperature, sarcomere shortening, ageing, and packaging	Weak	[16,41,53,57,61]
Meat color	Lightness, redness, yellowness, and chroma	Single born lambs may show greater redness in some studies, but the response is inconsistent	Age, myoglobin, diet, activity, pH, oxidation, and display conditions	Weak to moderate	[53,56,59,60,61]
Oxidative stability	Lipid oxidation, myoglobin oxidation, and discoloration	No established direction attributable to litter size	Fatty acids, vitamin E, plant antioxidants, packaging, and storage	Weak	[59,60,61,78,79,80]
Muscle development	Fiber number, fiber size, and myogenic capacity	Prenatal restriction may reduce fiber formation and alter later hypertrophy	Gestational stage, restriction severity, sex, genotype, and postnatal nutrition	Moderate for developmental effects	[39,40,42,85,86,87,88]
Tenderness	Shear force, sensory tenderness, and proteolysis	Usually unchanged after relevant slaughter variables are controlled	Slaughter age, intramuscular fat, connective tissue, chilling, stimulation, and ageing	Weak	[16,41,53,63,66]
Chemical composition	Moisture, protein, and intramuscular fat	Multiple born lambs may be leaner when slaughtered at a common age	Physiological maturity, sex, breed, diet, and slaughter weight	Moderate before adjustment and weak after adjustment	[16,52,53,54,63]
Fatty acid composition	Saturated, monounsaturated, and polyunsaturated fatty acids	No consistent response attributable solely to litter size	Maternal diet, finishing diet, forage species, rumen metabolism, and fatness	Weak	[74,75,76,77,78]
Flavor	Species flavor, pastoral flavor, and volatile compounds	Direct evidence for a litter size effect is scarce	Diet, age, skatole, indole, branched-chain fatty acids, and cooking	Insufficient	[70,74,82,83,84]
Consumer acceptance	Tenderness, juiciness, flavor, and overall liking	No demonstrated inherent disadvantage of meat from multiple-born lambs	Consumer background, cut, ageing, intramuscular fat, and production system	Insufficient	[63,64,70,71,72,73,83,84]
Product uniformity	Variation in weight, fatness, age, and eating quality	Larger litters may increase variation among lambs entering the slaughter group	Rearing type, milk supply, compensatory growth, drafting, and finishing strategy	Moderate at flock level	[4,10,15,46,52]

The reported directions represent the balance of available evidence and should not be regarded as universal responses. Multiple birth includes twins and larger litters, although most published comparisons combine triplets with other multiple-born lambs. Comparisons performed at a common age, live weight, carcass weight, or degree of fatness address different biological questions. The cited studies also differ in breed, sex, rearing type, feeding system, muscle, ageing duration, and analytical method. Nutritional restriction models provide mechanistic evidence and must not be pooled conceptually with natural multiple births. The citations for any row may include both direct birth-type comparisons and mechanistic or general meat-quality studies; the latter support plausible modifiers, not an independent causal effect of litter size. These endpoints should remain separate in interpretation: common age assesses development at a fixed time; common live weight retains possible differences in carcass yield and age; common carcass weight assesses comparable carcass size; and common fatness assesses a comparable finishing state. The qualitative evidence labels are not pooled effect estimates or a formal risk-of-bias grading.

**Table 4:** Minimum design and reporting framework for studies of litter size, carcass traits, and lamb meat quality.

Design Element	Minimum Requirement	Principal Bias Addressed	Core Records or Outcomes	References
Exposure definition	Record fetal number, birth type, and rearing type separately; document fostering and artificial rearing.	Confounding prenatal competition with postnatal milk access.	Birth weight, colostrum access, milk source, survival, and preweaning growth.	[10,26,27,28,45,46]
Experimental unit	Define the experimental unit before analysis; distinguish it from the lamb measurement unit. Account for litter/ewe dependence and sire, flock, year, and management-group effects where identifiable. Justify fixed or random treatment from sampling, available levels, and the target inference.	Pseudoreplication among littermates and underestimated uncertainty.	Ewe, sire, parity, sex, litter identity, and lamb identity.	[22,23,24,25]
Longitudinal follow-up	Link pregnancy, neonatal, growth, finishing, carcass, and meat records for the same identified lambs. Fit within-lamb correlation in longitudinal mixed models for repeated weights.	Attributing later outcomes to litter size without resolving intervening growth paths.	Repeated weight, health events, diet, age at slaughter, and carcass feedback.	[10,39,40,41,42,48,49]
Dual slaughter endpoints	Distinguish common-age, common-live-weight, common-carcass-weight, and common-fatness comparisons. Use complementary endpoints when feasible and justify covariates against the target effect; carcass weight may be a mediator.	Confounding developmental history with physiological maturity and carcass size.	Carcass weight, fat depth, muscularity, days to slaughter, and feed use.	[15,16,39,40,41,42,50]
Postmortem standardization	Standardize transport, fasting, chilling, ageing, packaging, muscle, and analytical protocols.	Masking or exaggerating birth-type effects through processing variation.	pH and temperature decline, purge or cooking loss, shear force, color, and oxidation.	[52,53,54,56,61,62]
Mechanistic phenotyping	Pair commercial traits with muscle structure, connective tissue, lipid composition, and metabolic indicators.	Inferring fetal programming from endpoint meat traits alone.	Fiber number and size, collagen, intramuscular fat, fatty acids, glycogen, and proteolysis.	[30,32,33,34,39,40,42,75,78,85,89]
Transparent reporting	Report group sizes for singleton, twin, and triplet lambs; effect estimates, uncertainty, exclusions, and covariate definitions.	Selective interpretation of small or unbalanced birth-type subgroups.	Adjusted and unadjusted estimates, confidence intervals, and prespecified contrasts.	[16,41,53]

The framework is intended for natural litter size comparisons and should not be treated as evidence that maternal nutrient restriction or experimental placental insufficiency is biologically equivalent to multiple pregnancy. Common-age and common-carcass-weight comparisons answer complementary questions. Ewe and litter effects should be included in the experimental structure, and birth type should be distinguished from rearing type. Models should account for correlated lambs within a litter and repeated weights within an individual; common-fatness endpoints may complement common-age and common-carcass-weight comparisons. Define the experimental unit before analysis and distinguish it from the measurement unit. Selection of fixed and random effects should reflect sampling, the number of factor levels, and the target inference. Carcass weight and birth weight may be mediators, so their adjustment requires an explicit biological rationale.

**Table 5:** Integrated strategies for balancing prolificacy, lamb survival, carcass output, and meat quality.

Intervention	Main Period of Application	Primary Objective	Expected Benefit	Potential Limitation	Recommended Outcome Measures	References
Balanced maternal selection index	Selection of replacement ewes	Combine prolificacy, maternal ability, survival, and ewe efficiency	Field and genetic association; expected selection response: Greater litter weight weaned and improved lifetime ewe output	Slow response for traits with low heritability	Lambs reared, litter weight, ewe longevity, and mature ewe weight	[94,95,96,97,98,99]
Balanced terminal sire selection	Ram selection	Improve growth, carcass yield, and carcass compliance	Genetic association; expected selection response: Fewer days to slaughter and more saleable meat	Excessive emphasis on leanness may reduce intramuscular fat	Growth rate, carcass weight, muscle depth, fatness, and intramuscular fat	[91,97,100,132,133,134]
Genomic and marker-assisted selection	Breeding program	Increase selection accuracy for reproductive and production traits	Genetic and genomic evidence; projected breeding benefit: Earlier and more accurate selection	Major prolificacy genes may produce excessive litter size	Genotype, ovulation rate, litter distribution, birth weight, and survival	[5,6,7,17,18,108,109]
Pregnancy scanning and fetal counting	Mid pregnancy	Match ewe nutrition and lambing management to fetal number	Field association and management evidence; expected benefit: More efficient feed allocation and improved lamb viability	Accuracy depends on operator and stage of pregnancy	Fetal number, BCS on a recorded scale, ewe live weight, birth weight, and survival	[45,110,111,112,113,114]
Targeted late pregnancy nutrition	Late pregnancy	Support fetal growth, colostrum production, and ewe health	Intervention evidence is context dependent; expected benefit: Improved birth weight and maternal supply; conditional on initial body condition and forage adequacy	Overfeeding single-bearing ewes can increase dystocia	BCS and its change, feed supply, colostrum yield, birth weight, and pregnancy toxemia	[45,110,112,113,118,119]
Smaller lambing groups and shelter	Lambing	Reduce interference, exposure, and mismothering	Field association; expected benefit: Improved ewe and lamb bonding and neonatal survival	Requires paddock capacity and additional management	Time to stand, time to suck, mortality cause, and survival	[28,104,114,116,117]
Early colostrum intervention	First hours after birth	Prevent starvation, hypothermia, and passive immune failure	Nutritional and behavioral evidence; expected benefit: Improved survival of small multiple-born lambs	Labor and disease transfer through pooled colostrum	Colostrum intake, body temperature, immunoglobulin status, and survival	[118,119,120,121,124]
Cross fostering and litter allocation	First day after birth	Match litter demand to ewe milk and maternal capacity	Management evidence; expected benefit: More uniform milk access and preweaning growth	Risk of rejection and disease transfer	Number reared, milk access, growth, and survival	[120,121,122,126]
Creep feeding and controlled weaning	Preweaning	Reduce dependence on ewe milk	Feeding and weaning intervention evidence; expected benefit: Improved growth and easier transition at weaning	Feed cost and variable intake response	Solid feed intake, weaning weight, and postweaning growth	[127,128,129]
Weight based grouping and targeted finishing	Postweaning	Reduce variation in slaughter readiness	Growth intervention evidence; expected benefit: More uniform carcasses and efficient feed use	Additional groups require facilities and labor	Growth rate, feed conversion, days to slaughter, and carcass compliance	[48,49,130,131]
Electronic identification and repeated weighing	Whole production cycle	Link prenatal history with growth and carcass outcomes	Proposed monitoring benefit, not an established production effect: Earlier detection of poorly performing lambs	Equipment cost and data management requirements	Individual growth curves, health events, and carcass feedback	[131,134]
Ultrasound and carcass feedback	Selection and finishing	Align live animal decisions with carcass value	Genetic and phenotypic evidence; expected benefit: Improved muscularity, fatness, and slaughter timing	Prediction accuracy varies among breeds and devices	Muscle depth, fat depth, carcass weight, conformation, and meat quality	[91,132,133,134]
Whole flock bioeconomic evaluation	Annual flock assessment	Identify the economically optimum litter size	Bioeconomic model output conditional on assumptions: Balances ewe output, production costs, survival, and carcass value; system-specific modeled expectation, not a universal optimum	Results are sensitive to prices and production environment	Revenue; feed, labor, veterinary and replacement costs; margin per ewe and per kg of saleable carcass; survival and carcass output	[3,4,94,97]

Expected responses are conditional on breed, ewe mature size, feed availability, climate, labor, and market requirements. Litter size at birth should be distinguished from rearing type and the number of lambs surviving to slaughter. Economic and environmental evaluation should use saleable carcass output rather than lambs born as the principal production endpoint. Expected benefits represent plausible or reported conditional responses rather than guaranteed commercial effects; the cited source and design should be consulted before interpreting a row as experimental evidence. The evidence qualifier at the start of each Expected benefit cell identifies the type of support; most entries are projected responses rather than effects demonstrated in a randomized commercial trial. The fostering row is supported by maternal-behavior and management evidence rather than a direct trial demonstrating the listed commercial benefit. Biological output, financial margin, and environmental burden should be reported separately. Environmental comparisons must state the functional unit and system boundary; biological improvement does not itself demonstrate economic or environmental improvement.

**Table 6:** Priority research questions for defining biologically and commercially optimum prolificacy.

Priority Question	Recommended Design	Primary Outcomes	Decision Value	References
What litter-size distribution maximizes saleable carcass output within a defined production system?	Prospective multi-flock cohorts or bioeconomic experiments stratified by breed, feed availability, climate, labor, and market endpoint. Specify lamb- or ewe-level estimands, litter/ewe clustering, and relevant flock effects.	Carcass weight and compliance per ewe exposed; ewe and lamb survival; feed, labor, and replacement costs.	Defines an optimum rather than a maximum litter size for each system.	[3,4,93,95,96,97]
How closely do maternal-restriction models reproduce natural twin and triplet pregnancy?	Direct comparison of natural singleton, twin, and triplet pregnancies with matched nutritional-restriction or placental-insufficiency models. Specify lamb- or ewe-level estimands, litter/ewe clustering, and relevant flock effects.	Placental function, fetal growth, endocrine status, muscle development, and neonatal vigor.	Separates evidence from complementary models from mechanisms specific to natural multiple pregnancy.	[26,27,28,30,31,39,40]
Can targeted postnatal nutrition normalize carcass readiness without altering tissue composition?	Randomized nutritional interventions within birth type, with repeated growth records and common-age plus common-carcass endpoints. Specify lamb- or ewe-level estimands, litter/ewe clustering, and relevant flock effects.	Feed efficiency, compensatory gain, muscle and fat deposition, slaughter age, and carcass uniformity.	Identifies when compensatory growth is biologically efficient and commercially useful.	[39,40,41,42,48,49,88,89]
Does litter size independently influence eating quality and shelf life?	Adequately powered singleton, twin, and triplet comparisons with standardized finishing, slaughter, chilling, ageing, and sensory testing. Specify lamb- or ewe-level estimands, litter/ewe clustering, and relevant flock effects.	pH, water-holding capacity, shear force, intramuscular fat, color stability, volatiles, and consumer scores.	Determines whether birth type adds predictive value beyond age, carcass weight, diet, and processing.	[16,41,52,53,59,61,63,64,70,74,84]
How should genomic information be integrated without producing unmanageable litter sizes?	Genomic evaluations that combine ovulation and litter traits with lamb survival, maternal ability, growth, carcass, and meat quality phenotypes. Specify lamb- or ewe-level estimands, litter/ewe clustering, and relevant flock effects.	Litter distribution, genotype dosage, lambs reared, ewe longevity, carcass value, and correlated responses.	Prevents selection for reproductive genotype from being disconnected from whole-system performance.	[5,6,7,17,18,95,108,109]
Can precision records improve welfare and flock-level efficiency in prolific systems?	Linked electronic identification, pregnancy scanning, repeated weighing, health events, ewe condition, and abattoir feedback across seasons. Specify lamb- or ewe-level estimands, litter/ewe clustering, and relevant flock effects.	Intervention timing, mortality causes, days to slaughter, carcass variability, and welfare outcomes.	Tests whether data-supported management converts additional fetuses into viable and marketable lambs.	[10,45,104,111,114,131,134]

Priorities should be evaluated at both lamb and ewe-levels by jointly analyzing saleable carcass output, survival, welfare, resource use, and product uniformity. References are representative and do not imply that every proposed design has already been implemented. For lamb-level outcomes, specify litter or ewe clustering and relevant sire, flock, and year effects; use longitudinal mixed models for repeated body weights. Define the experimental unit before analysis. Include sire, flock, year, and management-group effects where identifiable, with fixed or random treatment justified by the sampling structure, available levels, and inferential objective.

## Data Availability

No new data were created or analyzed in this study. Data sharing is not applicable to this article.

## Abbreviations

The following abbreviations and gene or allele symbols are used in this manuscript.

AMP	Adenosine monophosphate
*B4GALNT2*	Beta-1,4-N-acetyl-galactosaminyltransferase 2
BCS	Body condition score
*BMP15*	Bone morphogenetic protein 15
*BMPR1B*	Bone morphogenetic protein receptor type 1B
FecB	Booroola fecundity allele at *BMPR1B*
FecG	Fecundity allele designation associated with *GDF9*
FecGH	High-fertility allele at *GDF9*
FecLL	Lacaune fecundity allele at the *B4GALNT2* locus
FecXB	Belclare fecundity allele at *BMP15*
FecXG	Galway fecundity allele at *BMP15*
FecXH	Hanna fecundity allele at *BMP15*
FecXI	Inverdale fecundity allele at *BMP15*
FecXL	Lacaune fecundity allele at *BMP15*
FecXR	Rasa Aragonesa fecundity allele at *BMP15*
*GDF9*	Growth differentiation factor 9
*H19*	H19 imprinted, maternally expressed noncoding RNA gene
*IGF2R*	Insulin-like growth factor 2 receptor
S6	Ribosomal protein S6
